# Magnesium ions regulate the Warburg effect to promote the differentiation of enteric neural crest cells into neurons

**DOI:** 10.1186/s13287-024-04121-4

**Published:** 2025-01-23

**Authors:** Qiongqian Xu, Xixi He, Yaru Mou, Dong Sun, Xintao Zhang, Jichang Han, Xiaoyang Liu, Xingjian Liu, Xue Ren, Dongming Wang, Jian Wang, Chuncan Ma, Qiangye Zhang, Aiwu Li

**Affiliations:** 1 Department of Pediatric Surgery, Qilu Hospital of Shandong University, Jinan, China; 2 Department of Cardiology, Shandong Provincial Hospital Affiliated to Shandong First Medical University, Jinan, Shandong China; 3 Department of Pediatric Surgery, Xiangxi Tujia and Miao Autonomous Prefecture People’s Hospital, Xiangxi, China

**Keywords:** Magnesium Ions, Warburg effect, Enteric neural crest cells, Differentiation, Neurons

## Abstract

**Background:**

Understanding how enteric neural crest cells (ENCCs) differentiate into neurons is crucial for neurogenesis therapy and gastrointestinal disease research. This study explores how magnesium ions regulate the glycolytic pathway to enhance ENCCs differentiation into neurons.

**Materials and methods:**

We used polymerase chain reaction, western blot, immunofluorescence, and multielectrode array techniques to assess magnesium ions' impact on ENCCs differentiation. Non-targeted metabolomic sequencing, cellular acidification rate, oxygen consumption, and western blot analyzed sugar metabolism changes. D-glucose-^13^C6 isotope tracing identified key glucose flux changes. Surface plasmon resonance was used to detect the binding affinity of magnesium ions with key glycolysis genes. The elastic modulus of the hydrogel was measured using a universal testing machine, while pore size and porosity were assessed with scanning electron microscopy. Swelling ratios were determined using gravimetric analysis. In vivo, ENCCs in hydrogels were transplanted into renal capsule and subcutaneously, and magnesium ions' effects on ENCCs differentiation were evaluated.

**Results:**

Magnesium ions increased glycolysis levels during ENCCs differentiation into neurons, along with significant upregulation of neuronal markers β-Tubulin and ubiquitin C-terminal hydrolase L1, and enhanced functional neuronal properties. D-glucose-^13^C6 tracing results showed increased carbon flux in the glycolytic pathway after magnesium supplementation. The binding affinity of magnesium ions with the glycolytic key enzyme 6-phosphofructo-2-kinase/fructose-2,6-biphosphatase 3 was found to be 1.08 μM. Inhibiting glycolysis suppressed ENCCs differentiation into neurons, emphasizing its crucial role. The double-cross-linked hydrogel gelatin methacryloyl—alginate (gelMA—ALMA), cross-linked with magnesium ions, showed promise in enhancing ENCCs differentiation in vivo without causing systemic hypermagnesemia.

**Conclusion:**

Magnesium ions promote ENCCs differentiation into neurons by activating the Warburg effect. The GelMA-ALMA hydrogel serves as an effective localized magnesium delivery system, supporting neuronal differentiation in vivo.

**Graphical abstract:**

Magnesium ions target PFKFB3, enhancing glucose flux towards G3P and subsequent lactate production, while also promoting ENCCs differentiation into neurons by facilitating NAD+ generation, suppressing ROS, and maintaining mitochondrial homeostasis. Mg: Mg^2+^, Glu: glucose, LA: lactic acid. Ref to the creation software of the picture. 
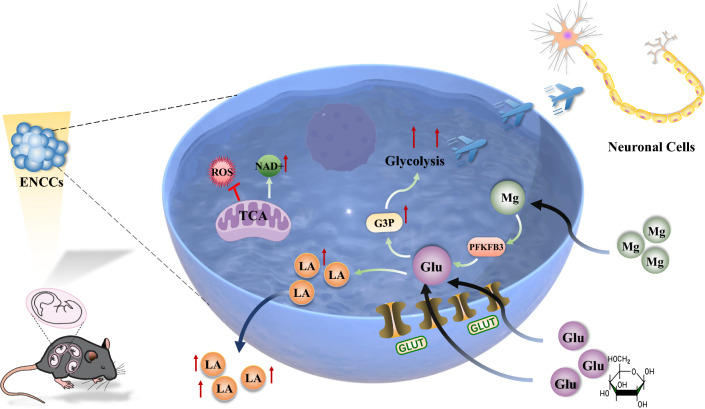

**Supplementary Information:**

The online version contains supplementary material available at 10.1186/s13287-024-04121-4.

## Introduction

The enteric nervous system (ENS), often dubbed the "second brain," intricately regulates intestinal functions. Originating from enteric neural crest cells (ENCCs), the ENS formation requires precise processes of survival, migration, proliferation, and differentiation within the gastrointestinal tract. Disruptions in these events may contribute to hirschsprung's disease (HSCR) [1–4]. Neural crest cells' founding population likely gets specified in the mid-stage of the original gut embryo formation. Extrinsic sensory neurons transmit signals from the intestine to the brainstem and spinal cord, essential for detecting various stimuli [5, 6]. The challenge of promoting neural crest cell differentiation into neurons, rather than glial cells, is a long-standing concern, and understanding the differentiation mechanism is crucial for unraveling diseases like HSCR.

Essential elements are vital for maintaining human body homeostasis, and magnesium homeostasis, in particular, is critical for cell survival. Disruptions in magnesium ions (Mg^2+^) transport can adversely affect *N* Methyl-D-Aspartate receptor function, leading to impaired synaptic plasticity and neuronal communication, which are associated with various neurological disorders [7]. Mg^2+^ also plays a role in the formation and maturation of neural networks, and disturbances in Mg^2+^ transport can potentially cause neurological abnormalities in neurodegenerative diseases like Alzheimer's disease. In Alzheimer's disease and other neurodegenerative conditions, Mg^2+^ has been shown to protect neuronal cells from cellular stress, increase the number of neural stem cells in the hippocampus, promote the differentiation of neural stem cells into neurons instead of glial cells in vivo, and may even ameliorate the pathology of Alzheimer's disease [8]. Furthermore, magnesium and its alloys have emerged as novel materials for repairing extensive nerve defects [9].

The Warburg effect represents a metabolic characteristic in cancer cells, characterized by elevated glycolysis that persists even in the presence of oxygen. During vertebrate embryo migration, stem cell populations undergo substantial metabolic changes before stratification, contributing to the Warburg effect. Enhanced glycolytic flux stimulates Yap/Tead signaling, triggering the expression of transcription factors and driving the transformation of epithelial cells into mesenchymal cells [10]. Additionally, numerous studies suggest that neurons metabolize glucose via glycolysis in vivo and rely on glycolysis for normal functionality [11]. In the brain, synaptic terminals are recognized as primary sites for Adenosine triphosphate (ATP) consumption, participating in action potential generation, vesicle release, and neurotransmitter recycling [12, 13]. Consequently, neuronal terminals efficiently generate ATP by engaging in glucose metabolism through oxidative phosphorylation (OXPHOS). Concurrently, synaptic plasticity enables terminals to overcome oxidative damage induced by reactive oxygen species (ROS) byproducts [14]. Conversely, oxidative stress in the soma, housing stored DNA and involved in lipid and protein synthesis, triggers irreversible damage to neurons [14–16]. Hence, a ROS-free aerobic glycolysis emerges as a more favorable metabolic strategy for the soma. Moderate aerobic glycolysis contributes to the antioxidant state of neurons [14]. However, despite Mg^2+^ acting as an essential cofactor for various enzymes in phosphate bond consumption, regulating key metabolic pathways like glycolysis and OXPHOS, and playing a vital role in cellular survival, there is limited research on the impact of glycolysis on enteric neurons. Additionally, there is a notable absence of studies on Mg^2+^ regulation of glycolysis in neural crest development and enteric neurogenesis.

The impact of elevated magnesium on the fate determination of neural cells during the development of enteric neural crest cells is still to be elucidated. Therefore, the primary purpose of this study is to explore the mechanism of Mg^2+^ action in regulating energy metabolism, promoting ENCCs differentiation into neural cells. Through in-depth research on the role of Mg^2+^, we hope to reveal critical mechanisms in the differentiation of intestinal neurons, providing new clues for the treatment of neurological diseases.

## Material and methods

### Cell culture

The C57BL/6J mice at gestational days 13–15 were euthanized by cervical dislocation, followed by immersion in 75% ethanol for 5 min and subsequent rinsing in sterile PBS to remove the ethanol. Embryos were aseptically removed, washed with PBS containing 1% penicillin–streptomycin, and then the gut tubes were dissected. The tissues were digested with 0.25% trypsin–EDTA solution at 37 °C for 20–30 min. The separated cells were filtered through a sterile cell strainer with a pore size of 70 μm, followed by centrifugation at 150g for 5 min. The collected cells were placed in culture flasks containing complete mouse enteric neural crest cell culture medium (CP-M216, PromoCell) and maintained at 37 °C with 5% CO_2_. The medium was half-changed every 24 h. Once the cells reached 80–90% confluence, they were passaged. After 2–3 passages, the expression of anti-P75 (17–9400-42, Invitrogen) was assessed using a BD FACS Calibur flow cytometer (BD Biosciences, NJ, USA), confirming that over 90% of the cells expressed this marker (Figure Supplementary 1), making them suitable for subsequent experiments. Neuronal differentiation medium consisted of a 1:1 mix of Neurobasal (Gibco, 21103–049) and DMEM/F12 (Gibco, C11330500BT) media supplemented with N2 (Gibco, 17502048), B27 (Gibco, 17504-044), L-glutamine, and β-mercaptoethanol in the presence of Bmp4 (peprotech; 315-27-10UG; 20 ng/ml), Gdnf (peprotech; 450-44-10UG; 20 ng/ml), and bFgf (peprotech; 450-33-50UG; 20 ng/ml) [17].

### Transfection

ENCCs were cultured and seeded in a 6-well plate. The cell density in each well was 2 × 10^5^, and each well was filled with 2 ml of mouse ENCCs culture medium. When cells covered approximately 80% of the well, transfection was performed using mouse 6-phosphofructo-2-kinase/fructose-2,6-biphosphatase 3 (PFKFB3) plasmid DNA (GeneChem, China) and Lipofectamine 2000 (Thermo Fisher) according to the manufacturer's instructions. Transfection efficiency of plasmids was assessed 48 h later using qRT-PCR.

### Measurement of cellular metabolism by seahorse

Eight days before the Seahorse XF experiment, seed 100,000 ENCCs per well in the Seahorse XF96 cell plate. On the second day, replace the medium with neuron cell differentiation medium containing or lacking 10 mM MgCl_2_. One day before the experiment, hydrate the sensor plate with 200 μL of sterile water, incubate it in a 37 °C, non-CO_2_ cell culture incubator overnight, and preheat the Seahorse XFe96 assay system at 37 °C for at least 5 h. Replace sterile water in the sensor plate with Seahorse XF calibration solution and switch the growth medium in the cell plate with Seahorse XF assay medium during the experiment. Conduct a glycolysis stress test using an analysis solution without glucose. Measure extracellular acidification rate (ECAR) every 5 min before and after the sequential introduction of glucose, oligomycin, and 2-DG. Assess cellular oxygen consumption rate (OCR) with the Mito Stress Test reagent kit. This Seahorse XF Analyzer-based methodology provides a comprehensive understanding of cellular metabolic dynamics in ENCCs under different magnesium conditions.

### Surface plasmon resonance (SPR) experiment

To evaluate the affinity of biotin-labeled PFKFB3 nucleic acid for magnesium ions, a Biacore T200 instrument (GE Healthcare, Uppsala, Sweden) is used. SA sensor chips immobilize the nucleic acid, which is coupled to the chip using the biotin coupling method. Before immobilization, the chip surface is washed with a mixture of 50% isopropanol, 50 mM NaOH, and 1 M NaCl. The experiment is conducted at 25 °C using a buffer solution containing 50 mM NaOH and 1 M NaCl, with 1.05 × HBS-P + 5% DMSO running buffer at pH 7.4. Experimental steps include baseline measurement, association phase (flowing the analyte, biotin-labeled PFKFB3, over the immobilized nucleic acid), and dissociation phase (monitoring the dissociation of the bound analyte from the nucleic acid). Data normalization is done to adjust the y-axis, making the sensor response 100 relative units (RUs). The Biacore T200 evaluation software (version 3.0) is used to assess the affinity of magnesium ions for biotin-labeled PFKFB3 nucleic acid by measuring the equilibrium dissociation constant (KD).

### Ultra performance liquid chromatography–mass spectrometer (UPLC–MS)

To analyze water-soluble metabolites in ENCCs undergoing neuronal differentiation, 1 × 10^7^ ENCCs were cultured in a 10 cm dish with neuronal differentiation medium for seven days. After cellular metabolism arrest on ice, cells were washed with PBS and extracted with 600 μL of methanol. Metabolite analysis was performed using a Waters UPLC I-Class Plus system coupled with a Q Exactive high-resolution mass spectrometer. Chromatographic separation utilized a BEH C18 column at 45 °C with a flow rate of 0.35 mL/min. Mass spectrometry was conducted in both positive and negative ion modes, using appropriate mobile phases and gradients. Key parameters included a primary scan range of 70–1050 m/z and a resolution of 70,000.

Data analysis was carried out using Compound Discoverer 3.3 software with integrated databases (BGI Metabolome Database, mzCloud, ChemSpider). Differential metabolites were identified based on variable importance in projection (VIP) ≥ 1, fold change ≥ 1.2 or ≤ 0.83, and *p*-value < 0.05. Hierarchical clustering of normalized metabolite expression levels was conducted to compare metabolic flux under different conditions, using six replicates per experimental group. This approach allowed precise evaluation of metabolite labeling dynamics and metabolic flux in ENCCs under different magnesium conditions, with each experimental group comprising 6 replicate samples.

### Labelled metabolites of isotopomers measurement

ENCCs were seeded at a density of 1 × 10^7^ cells per 10 cm dish and cultured in neuronal differentiation medium with or without magnesium for 7 days. On the seventh day, the medium was replaced with neuronal differentiation medium containing 50% D-glucose-^13^C6 (389374, Sigma) and 50% D-glucose (50–99-7, Macklin) to initiate stable isotope labeling experiments lasting 8 h.

At the end of the labeling period, the supernatant was removed, and all samples were placed on dry ice to halt metabolism. Metabolites were extracted with 600 μL of methanol, and water-soluble metabolites were analyzed using the Q Exactive Plus mass spectrometer. Mass spectrometry scans were conducted in negative ion mode at a resolution of 140,000 (m/z 200), with an automatic gain control target of 1 × 10^6^ and a scan range of m/z 75–1000. Isotope labeling data were analyzed using El-MAVEN with natural abundance correction. This approach enabled a precise assessment of metabolite labeling dynamics and metabolic flux in ENCCs under varying magnesium conditions, with each experimental group comprising 4 replicate samples (Table 1).

**Table 1:** The primers for the target genes

Target gene	Forward primer sequence	Reverse primer sequence
β-actin (Mus)	CCATGTACGTAGCCATCCAG	CGTTGCCAATAGTGATGACC
TUJ1 (Mus)	CAGAGCCATTCTGGTGGAC	GCCAGCACCACTCTGACC
UCHL1 (Mus)	ATTCAGGCAGCCCATGACT	GAAATTCACTTTGTCGTCTACCC
PFKFB3 (Mus)	CGCAATAGTGTCACCCCACT	CATGTTTTGTCCGGGCAGC
HK-II (Mus)	AGGCTACCCGGAGTTGTTCT	TCCCGTCGCTAACTTCACTC
LDHA (Mus)	GCTTCCATTTAAGGCCCCGC	GGTCCTTGAGGGTTGCCATC
GLUT1(Mus)	GGCGGCGGTCCTATAAAAAG	ACGGACGCGCTGTAACTATG

### Microelectrode array (MEA) electrophysiology

To prepare the culture plate, coat it overnight with 10 ng/ml lysine, and then wash it five times with PBS. Adjust the cell concentration to 10,000 cells in 10μL and seed onto the Recording electrodes area. Incubate at 37 °C with 5% CO_2_ for one hour. Add 250 µl of enteric neural crest cell culture medium by tilting the plate along the well wall to avoid disrupting the cell droplets. Pierce the droplets with a pipette tip and add the remaining medium. After 24 h, switch to neuron-inducing culture medium and continue cultivation for 14 and 28 days. Perform electrophysiological activity detection using the Maestro HT MEA System. The MEA chip interfaces with an optimized data acquisition system for recording neuronal electrical signals. AxISNavigator collects data, and neural metric tool analyzes pulse patterns, frequency, and synchrony. Quantitative results visualize neural network dynamics, aiming to elucidate differences in electrophysiological activity during ENCCs differentiation into neuronal cells under varying magnesium conditions.

### Preparation and characterization of microgel composite hydrogel

For the microgel composite hydrogel preparation, MgCl_2_ is dissolved in deionized water to create a 10 mM solution, filtered with a 0.22 μM filter. 12% (w/v) gelatin methacryloyl (gelMA) (EFL-GM-30, China) and 6% (w/v) alginate methacryloyl (ALMA) (EFL-AlgMA-50 K, China) are separately prepared using deionized water and the MgCl_2_ solution. These formulations are freeze-dried for 2 h, cryogenically fractured with liquid nitrogen, and observed under a scanning electron microscope. To test dissolution, the pre-weighed gel (M0) is immersed in PBS at 37 °C for 24 h. After removal, the hydrogel is reweighed (M1), and the swelling ratio is calculated using the formula (M0-M1)/M0*100%. The elastic modulus is determined using a universal testing machine (Shimadzu AGS-X-50N, Japan). This approach provides insights into the structural and solubility characteristics of the microgel composite hydrogel.

### In vivo ectopic transplantation of ENCCs encapsulated in gelMA-ALMA hydrogel

The work has been reported in line with the ARRIVE guidelines 2.0 [18]. 6-week-old male Sprague–Dawley (SD) rats were obtained from SPF (Beijing) Biotechnology Co. Ltd. and randomly divided into two groups, with 5 rats in each group. GelMA hydrogel at 12% concentration, ALMA hydrogel at 6% concentration, and ENCCs were prepared with or without MgCl_2_ encapsulation. After 1-min ultra violet curing, the gelMA-ALMA-ENCCs constructs were cultured in ENCCs medium. Anesthesia was induced with a 2% 30 mg/100 g intraperitoneal injection of sodium pentobarbital. Rats were fasted for 12 h prior to surgery, with access to water allowed. Under sterile conditions, the back skin in the kidney region was shaved and disinfected. A longitudinal incision approximately 2 cm lateral to the spine was made on the lateral abdomen, and the skin and muscle layers were carefully dissected to expose the kidney. A small incision (0.1–0.3 cm) was made in the perirenal fat capsule, avoiding damage to the kidney parenchyma. The GelMA-ALMA hydrogel containing ENCCs was inserted into the capsule through this incision. The renal capsule, muscle layers, and skin were then sutured in sequence.

To ensure adequate perioperative pain management, meloxicam (1–2 mg/kg, subcutaneously) was administered preoperatively as a preemptive analgesic, followed by additional doses every 24 h for 3 days post-surgery. Blood samples (500 μL) were collected from the rat tail vein at 0, 1, 4, 7 and 10 days post-transplantation for inductively coupled plasma mass spectrometry analysis of Mg^2+^ and Ca^2+^ concentrations. This assesses whether gelMA-ALMA-Mg is released into the bloodstream, potentially causing hypermagnesemia. At 6 and 12 weeks post-transplantation, ENCCs differentiation was assessed using the MEA system for electrophysiological activity and IF for neural marker. The rats were euthanized using high concentrations of carbon dioxide after the experiment. This methodology aims to elucidate the in vivo effects of magnesium-ion-loaded gelMA-ALMA hydrogel on ENCCs differentiation into neuronal cells, providing insights into the regenerative potential of this hydrogel system.

#### Statistical analysis

Statistical analysis was performed using GraphPad Prism 9 software (GraphPad, USA). Data are expressed as mean ± standard error of the mean (SEM). The normality of the data was confirmed using the Shapiro–Wilk test. A significance level of *p* < 0.05 was used to indicate significant differences. For data that passed the normality test, Student's t-test or one-way analysis of variance followed by Tukey's post hoc test for multiple comparisons was used. For data that did not pass the normality test, the Mann–Whitney U test was used.

## Results

### Magnesium ions facilitate the differentiation of ENCCs into neuronal cells in vitro

ENCCs were subjected to a gradient concentration (1–100 mM) treatment of MgCl_2_ in neuronal induction culture medium for 48 h. Notably, the total apoptosis rate (late + early stages) of ENCCs was lowest at 10 mM MgCl_2_ (Supplementary Fig. 2A, B). Cell viability, as assessed by CCK-8 results within the 1–100 mM range, consistently exceeded that of the negative control (NC) group, indicating that magnesium ions within this range did not hinder the proliferation and survival of ENCCs. Significantly, the OD value was highest at 10 mM MgCl_2_, suggesting optimal cellular vitality at this concentration (Supplementary Fig. 2C). Given the observed favorable proliferation and low apoptosis rate at 10 mM, we selected this concentration as the optimal Mg^2+^ concentration to promote ENCCs differentiation into enteric neurons. Due to the lack of a universally recognized drug for promoting the differentiation of ENCCs into neurons, a positive control was not included in this experiment.

**Fig. 1:**
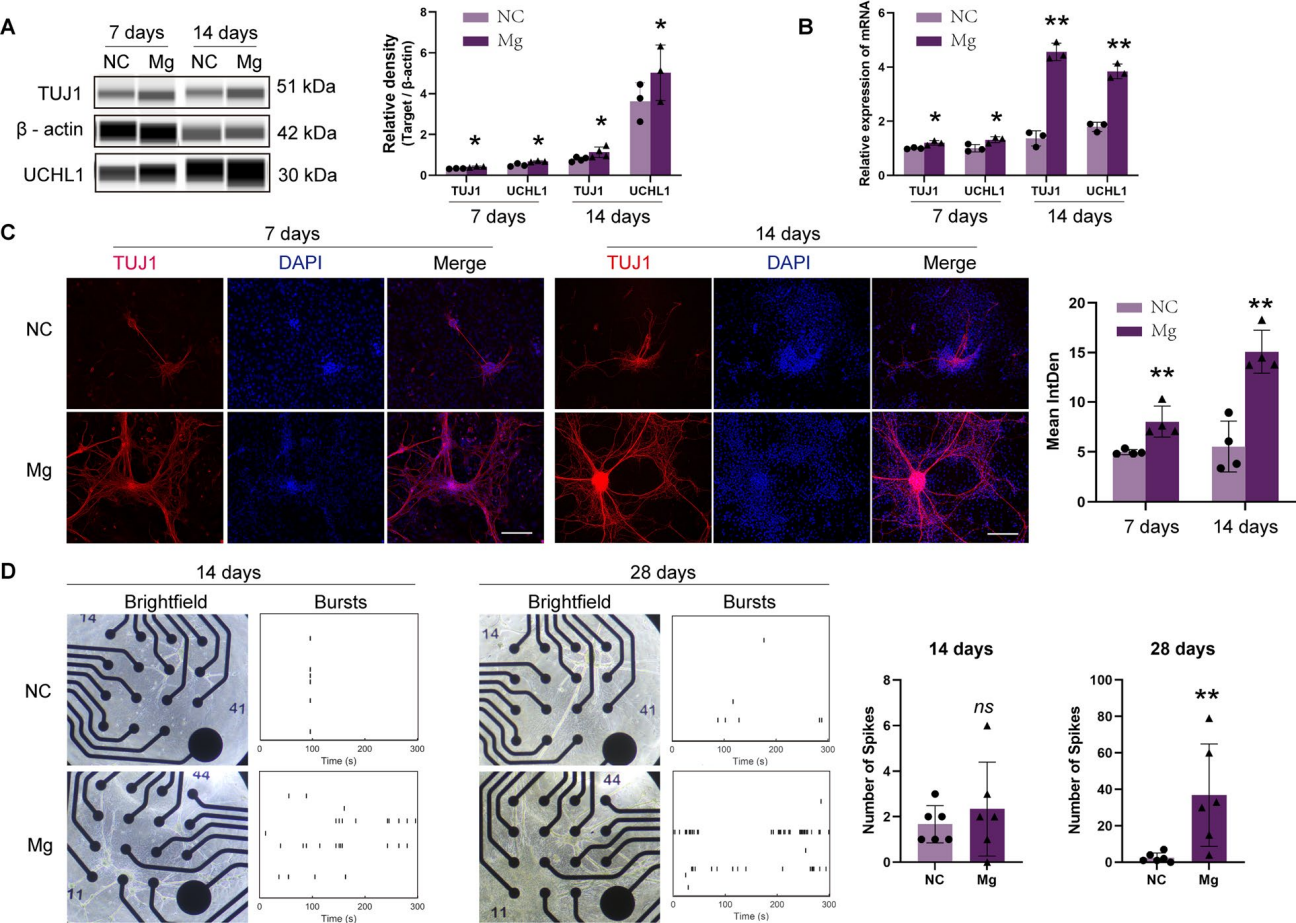
Magnesium-rich environment promotes ENCCs differentiation into neurons with exhibited electrical activity. **A**–**B** Western blot and polymerase chain reaction analyses demonstrate a significant increase in neuronal markers TUJ1 and UCHL1 in ENCCs cultured for 7 and 14 days in a high magnesium environment, proportional to the culture duration (n = 3) (full-length blots/gels are presented in Supplementary Material 16A, 16B). **C** Immunofluorescence shows that ENCCs can differentiate into neurons under both magnesium-containing and magnesium-free conditions, with higher TUJ1 expression and more pronounced axons observed in high magnesium conditions (n = 4). Scale bars: 170 μm. **D** ENCCs cultured for 14 and 28 days in neurogenic induction media with or without magnesium exhibit increased axon formation under bright-field microscopy, and microelectrode array results show more spikes in high magnesium conditions (n = 6). NC: negative control, Mg: Mg^2+^. Data were presented as mean ± SEM. *p < 0.05; **p < 0.01

When ENCCs were exposed to elevated magnesium conditions, the protein and gene expression levels of the neuronal markers TUJ1 and UCHL1 significantly increased (Fig. 1A, B), underscoring the critical role of increased Mg^2+^ concentration in promoting ENCC differentiation into neuronal lineages. Immunofluorescence analysis showed that ENCCs could differentiate into neurons under both magnesium-rich and magnesium-free conditions. Notably, higher TUJ1 expression and more pronounced axonal development were observed under elevated magnesium conditions (Fig. 1C).

To explore the functional neuronal characteristics of differentiated cells, we employed MEA to detect their electrophysiological activity. ENCCs exposed to elevated Mg^2+^ concentrations exhibited typical neuronal electrical signals (Fig. 1D). These findings not only affirmed the successful differentiation of ENCCs into neuronal cells but also highlighted their acquisition of functional neuronal attributes in response to heightened Mg^2+^ concentrations.

### Magnesium ions promote the differentiation of ENCCs into neuronal cells through the Warburg effect

**Fig. 2:**
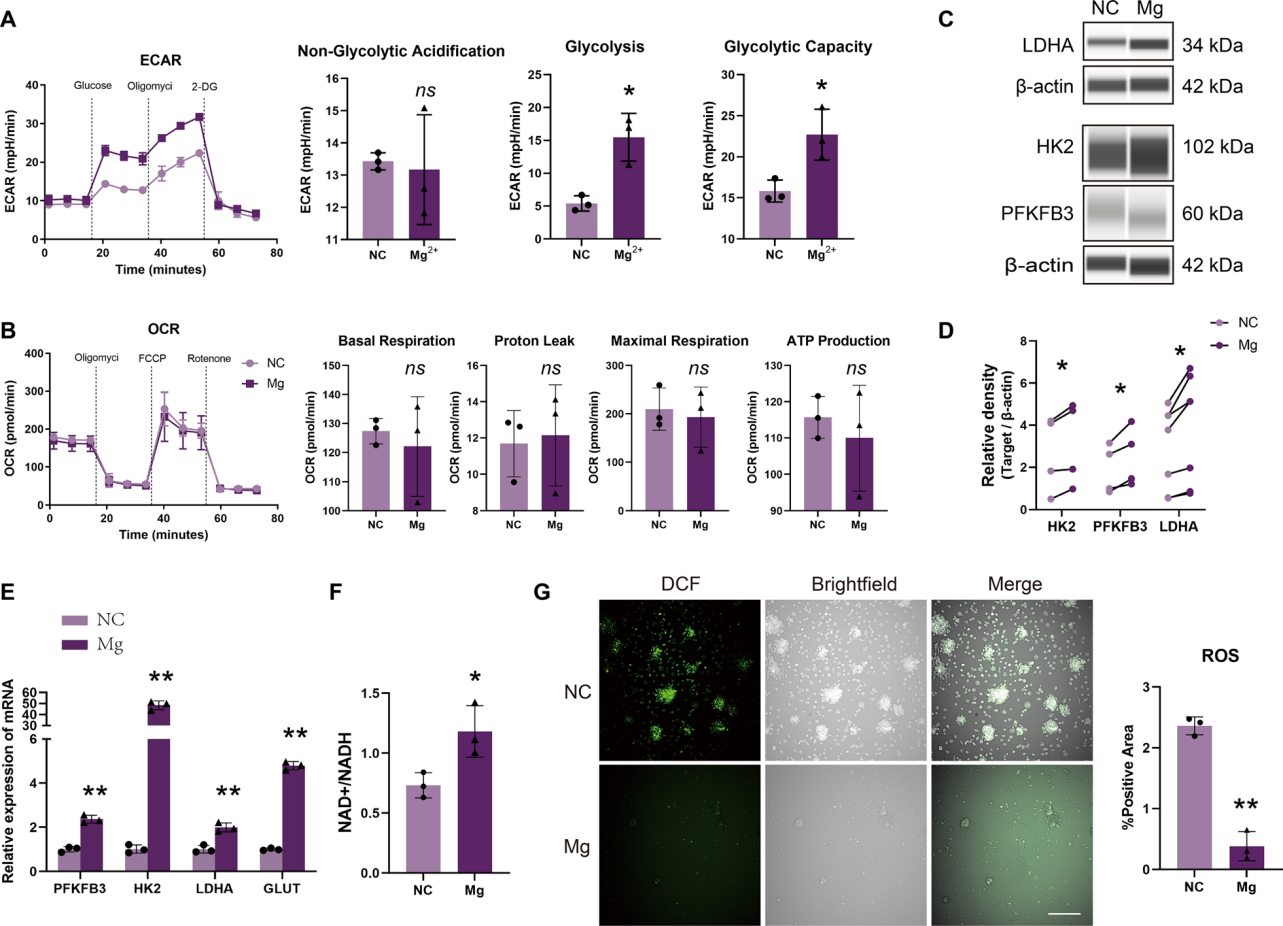
High magnesium environment activates the ENCCs Warburg effect. **A**–**B** ENCCs show no significant changes in non-glucose metabolism before and after magnesium supplementation. Mg^2+^ group exhibits significantly upregulated glycolysis and glycolytic capacity ECAR compared to the non-supplemented group. However, OCR values for basal respiration, proton leak, maximal respiration, and ATP production in mitochondrial stress testing show no significant changes (n = 3). **C**–**D** Immunoblotting reveals upregulation of glycolysis markers HK2, PFKFB3, and LDHA in high magnesium conditions (n ≥ 4) (Full-length blots/gels are presented in Supplementary Material 16C). **E** PCR shows increased mRNA levels of glycolysis markers HK2, PFKFB3, LDHA, and GLUT in high magnesium conditions (n = 3). **F**–**G** After magnesium supplementation, NAD + /NADH ratio increases, and mitochondrial ROS decreases (n = 3). Scale bars: 170 μm. ECAR: extracellular acidification rate, OCR: oxygen consumption rate. NC: negative control, Mg: Mg^2+^. Data were presented as mean ± SEM. *p < 0.05; **p < 0.01

Seahorse analysis has unveiled the regulatory role of Mg^2+^ in cellular metabolism. Throughout the 7-day process of ENCCs differentiation into neurons, the glycolytic stress assay results indicated that Mg^2+^ do not impact non-glycolytic acidification but instead elevate both glycolysis ECAR and glycolytic capacity ECAR in the cells (Fig. 2A). Conversely, mitochondrial stress results demonstrated that the increased presence of Mg^2+^ does not affect basal respiration, maximal respiration, or ATP production in the cells (Fig. 2B). In other words, the addition of Mg^2+^ does not alter the OCR values of the tricarboxylic acid (TCA), thereby preserving the original state of the mitochondria. This suggests that, upon the introduction of Mg^2+^, ENCCs primarily depend on the glycolytic pathway for energy during this stage of differentiation, rather than relying on the TCA pathway. For a more in-depth understanding, PCR and WB further validated a significant increase in key glycolytic enzymes, including GLUT1, HK2, PFKFB3, and lactate dehydrogenase A (LDHA), under high magnesium conditions. This reinforces the notion that energy during this stage is predominantly supplied by glycolysis (Fig. 2C–E). Furthermore, the elevated levels of Mg^2+^ lead to increased NAD + production, decreased intracellular ROS, and maintenance of mitochondrial homeostasis throughout the differentiation process (Fig. 2F, G).

To explore the metabolic changes induced by Mg^2+^ during the differentiation of ENCCs into neurons, we conducted untargeted metabolomic profiling. A total of 560 metabolites were identified, with 59 upregulated metabolites and 11 downregulated metabolites. The heatmap illustrates that the differentially expressed metabolites are predominantly upregulated, with the upregulated metabolites primarily belonging to various amino acids (Supplementary Fig. 3). This indirectly suggests that under the influence of Mg^2+^, ENCCs' metabolic capacity is enhanced. This indicates that Mg^2+^ activate the Warburg effect, promoting ENCCs' differentiation into neuronal cells.

### Magnesium ions promote carbon flux

**Fig. 3:**
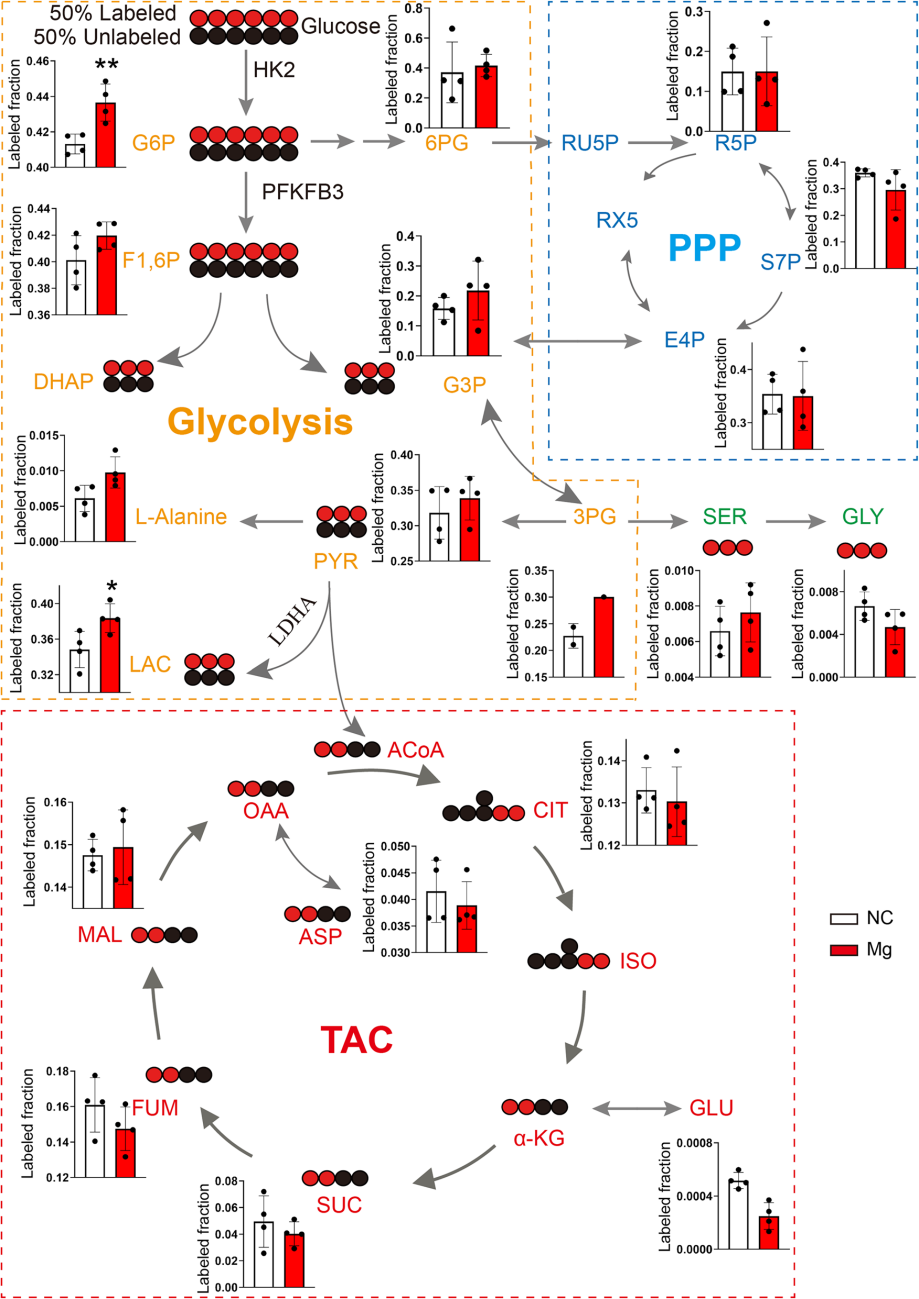
Carbon flux of D-glucose-^13^C6 in glycolysis, PPP, and TCA cycle in high magnesium environment. This schematic demonstrates increased carbon flux in the glycolysis pathway, leading to elevated lactate production in ENCCs in a high magnesium environment. However, there is no significant change in carbon flux in the PPP and TCA pathways (n = 4). PPP: Pentose Phosphate Pathway, TCA: Tricarboxylic Acid. NC: negative control, Mg: Mg^2+^. Data were presented as mean ± SEM. *p < 0.05; **p < 0.01

To further investigate changes in glucose metabolism flux during ENCCs differentiation into neurons under the influence of Mg^2+^, this study employed D-glucose-^13^C6 to trace carbon flux changes as ENCCs differentiated into neurons. After an 8-h tracking period, metabolites were extracted using methanol. Starting from glucose, the metabolic rate of glycolysis in the Mg 2 + group cells was significantly higher than that in the NC group, consistent with the glycolytic stress results observed in Seahorse analysis (Fig. 2A). From the metabolic flux results, it is evident that the Glucose-6-Phosphate (G6P) flux in the magnesium-treated group is significantly higher than that in the NC group, accompanied by the substantial production of lactate. This indicates that glycolysis is significantly higher in the magnesium-treated group than in the NC group. Additionally, a significant amount of Glyceraldehyde-3-Phosphate (G3P) is produced; however, there is no significant difference between the Mg 2 + and NC groups when G3P enters the Pentose Phosphate Pathway (PPP). G3P is then metabolized into 1,3-Bisphosphoglycerate (3GP), which is further converted into pyruvate (PYR). A portion of PYR generates lactate, which is excreted, while another portion of PYR enters the tricarboxylic acid (TCA) cycle under the action of acetyl coenzyme A. However, there is no significant difference in the TCA cycle between the Mg and NC groups. These results suggest that during the differentiation of neural crest cells into neurons, magnesium primarily enhances cellular energy production through the glycolytic pathway, rather than through the PPP pathway and TCA pathway, (Fig. 3).

### Inhibiting glycolysis suppresses the differentiation of ENCCs into neurons

**Fig. 4:**
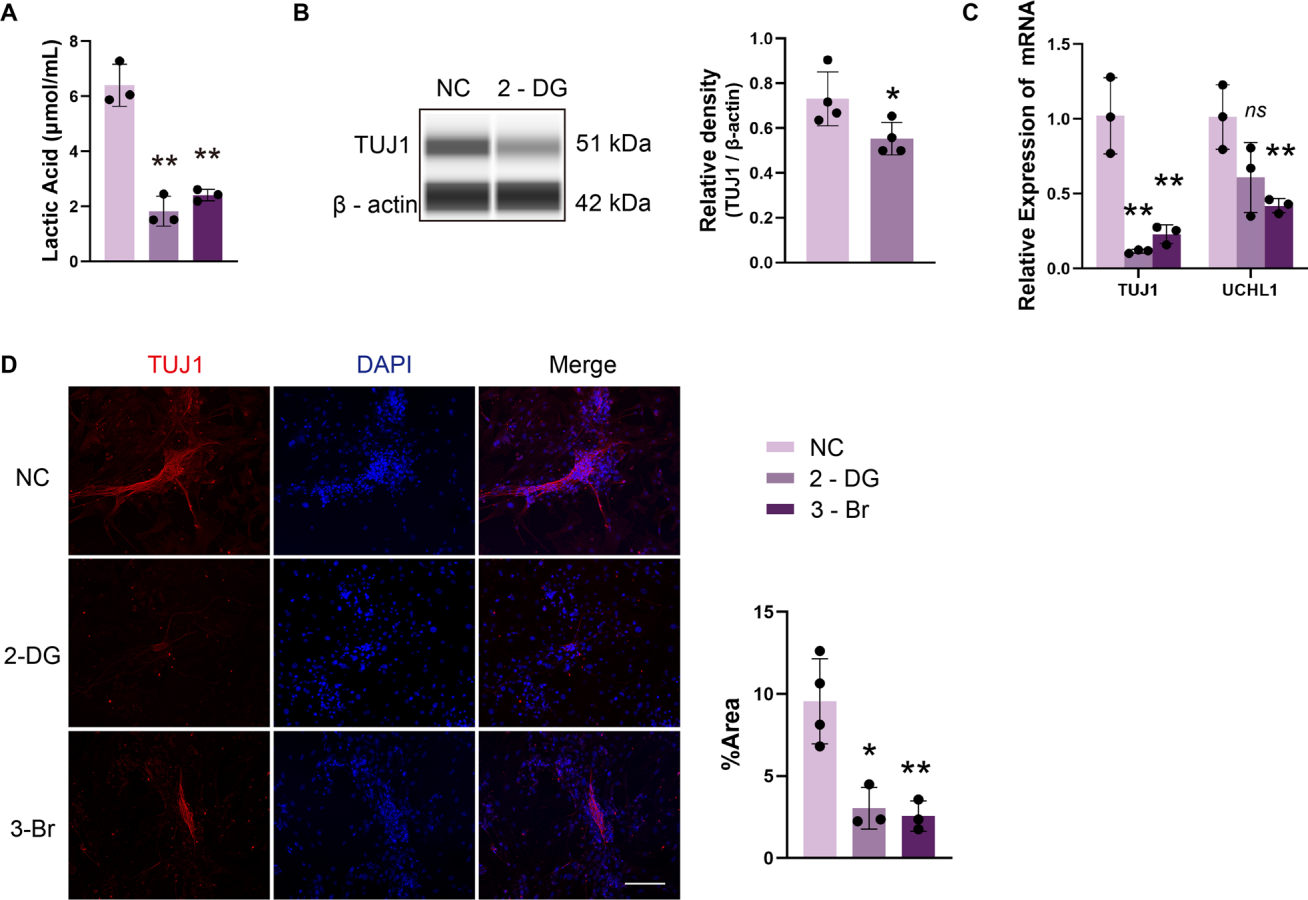
Inhibition of glycolytic pathway suppresses ENCCs differentiation into neurons. **A** Lactate levels significantly decrease after adding 2-DG and 3-Br to ENCCs (n = 3). **B** Immunoblotting shows a significant decrease in TUJ1 protein levels after culturing ENCCs with 2-DG for 7 days (n = 4) (Full-length blots/gels are presented in Supplementary Material 16D). **C** PCR indicates a significant decrease in TUJ1 and UCHL1 mRNA levels after adding 2-DG and 3-Br to ENCCs (n = 3). **D** Immunofluorescence shows a significant reduction in fluorescence area after adding 2-DG and 3-Br to ENCCs (n ≥ 3). Scale bars: 170 μm. NC: negative control. Data were presented as mean ± SEM. *p < 0.05; **p < 0.01

To validate the hypothesis that inhibiting glycolysis can impede the differentiation of ENCCs into neurons, this study utilized 2-deoxy-D-glucose (2-DG) as a glycolysis inhibitor [10]. The results revealed a decrease in the protein and mRNA levels of TUJ1 and UCHL1 following the addition of 2-DG. After treating ENCCs with 2-DG for one week, there was a reduction in lactate production (Fig. 4A). Significantly, the inhibition of glycolysis exerted a notable impact on ENCCs differentiation into neurons. Treatment with 2-DG resulted in diminished expression levels of neuronal-specific markers TUJ1 and UCHL1 at both the protein and RNA levels compared to the NC group (Fig. 4B–D). Furthermore, similar results were observed with the addition of 3-Bromopyruvate (3-BP). Thus, a high glycolytic flux is deemed essential for the efficient differentiation of neural crest cells into neurons.

### Mg.^2+^ regulates the ENCCs glycolysis pathway by targeting PFKFB3

Fructose-1,6-bisphosphatase (PFK) 1 catalyzes the conversion of fructose-6-phosphate (F6P) to fructose-1,6-bisphosphate (FBP), a crucial step in determining the glycolytic flux. PFK1 undergoes allosteric regulation by several metabolites, including ATP and fructose-2,6-bisphosphate (f-2,6-bp), which is a diversion product from PFK2 to F6P. PFK2, a well-known bifunctional enzyme with both kinase and phosphatase activities, indicates that the enzyme catalyzes the formation and degradation of f-2,6-bp. PFK2 has four isoforms, namely PFKFB1, PFKFB2, PFKFB3, and PFKFB4, each exhibiting tissue-specific expression patterns and different kinase-to-phosphatase activities. PFKFB3, with the highest kinase activity, guides glucose towards the glycolytic pathway (highest kinase: phosphatase ratio 710:1) (19, 20).

**Fig. 5:**
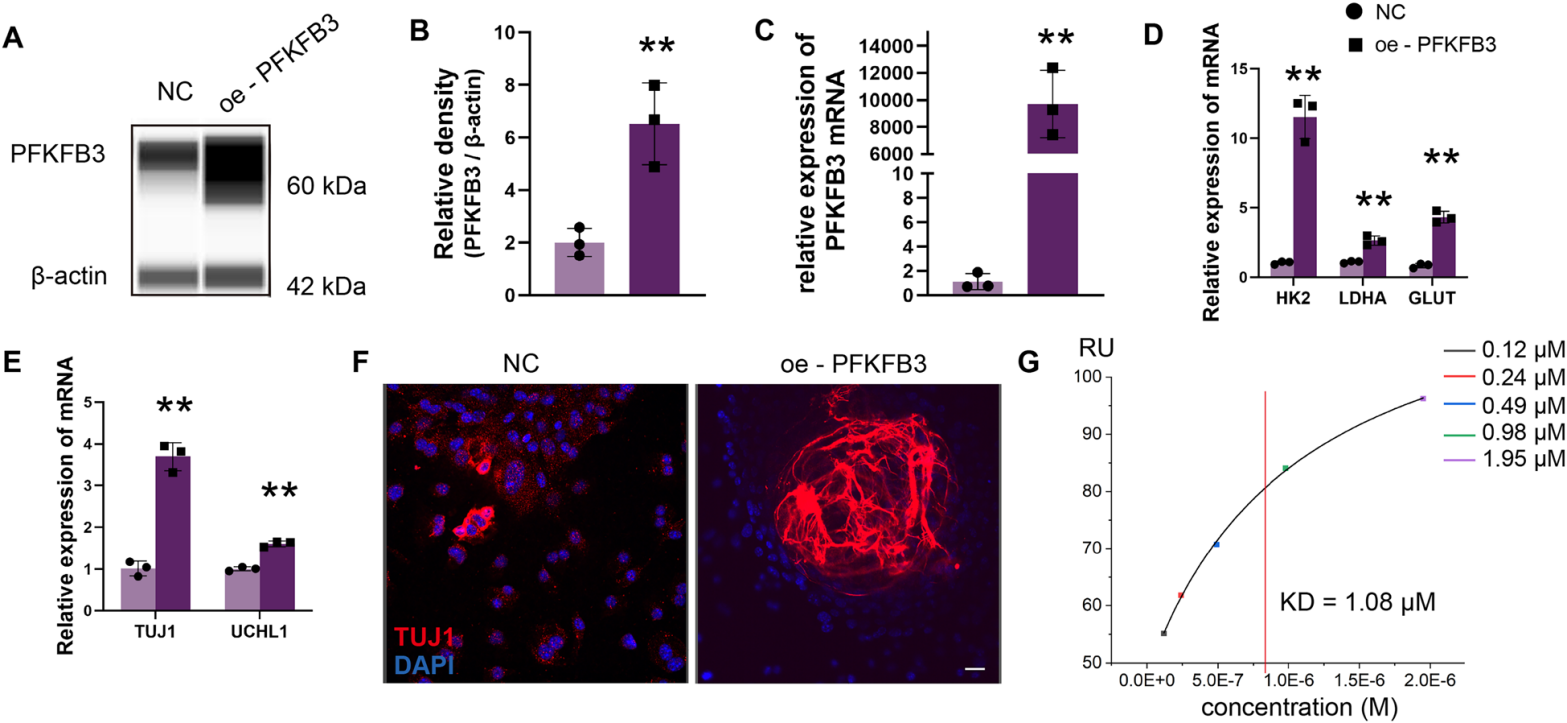
Magnesium ions target PFKFB3 to regulate glycolytic metabolism, promoting ENCCs differentiation into neurons. **A**–**C** WB and PCR confirm the efficiency of ENCCs overexpression of PFKFB3 (n = 3) (Full-length blots/gels are presented in Supplementary Material 16E). **D** Overexpression of PFKFB3 in ENCCs leads to increased expression of HK2, LDHA, and GLUT (n = 3). **E**, **F** Overexpression of PFKFB3 in ENCCs using neurogenic induction media for 7 days results in a significant upregulation of TUJ1 and UCHL1 (n = 3). **G** Surface plasmon resonance results show a strong binding relationship between magnesium ions and biotin-labeled PFKFB3, with a kDa of 1.08 μM. NC: negative control. Data were presented as mean ± SEM. *p < 0.05; **p < 0.01

To verify the role of PFKFB3 in ENCCs differentiation into neurons, PFKFB3 overexpression was performed (Fig. 5A–C). The mRNA expression levels of glycolysis-related genes HK2, LDHA, and GLUT increased (Fig. 5D), along with upregulated expression of neuronal markers TUJ1 and UCHL1 (Fig. 5E, F). Given that earlier results demonstrated the ability of Mg^2+^ to promote ENCCs glycolytic energy, to verify whether Mg^2+^ could bind to key glycolytic enzymes PFKFB3, we conducted further validation using surface plasmon resonance. The results confirmed that Mg^2+^ exhibited an affinity of 1.08 μM with biotin-labeled PFKFB3, with a chi-square value of 0.31, indicating a strong binding relationship between Mg^2+^ and biotin-labeled PFKFB3 (Fig. 5G). These results indicate that Mg^2+^ target PFKFB3 to regulate glycolytic metabolism, thereby promoting ENCCs differentiation into neurons.

### Performance evaluation of hydrogel bio-scaffolds

In pursuit of enhancing the differentiation of ENCCs by establishing a locally elevated magnesium environment, we synthesized a novel dual-crosslinked hydrogel using photocrosslinkable gelMA and ALMA (Figure Supplementary 4A, B). This innovative hydrogel was developed to encapsulate high concentrations of Mg^2+^ while effectively immobilizing cells within its matrix. Notably, scanning electron microscopy (SEM) observations revealed the formation of gels for both gelMA, ALMA, and gelMA + ALMA, with gelMA-ALMA displaying a superior and uniform pore structure compared to ALMA and gelMA (Figure Supplementary 4C, D). Furthermore, the elastic modulus of gelMA-ALMA was found to be higher than that of either gelMA or ALMA alone (Figure Supplementary 4E, F). Importantly, the gelMA-ALMA hydrogel exhibited lower swelling rates than individual gelMA and ALMA (Figure Supplementary 4G). This dual-crosslinked hydrogel demonstrates promising characteristics for creating a localized magnesium-rich microenvironment. Its superior pore structure, reduced swelling rates, and enhanced elastic modulus highlight its potential as an effective carrier for ENCCs, facilitating their differentiation.

Our results distinctly indicate that the hydrogel promotes ENCCs proliferation in vivo, emphasizing its biocompatibility and potential to enhance cell growth in a magnesium-rich microenvironment. In conclusion, the innovative gelMA-ALMA dual-crosslinked hydrogel provides a promising strategy for promoting ENCCs differentiation in a locally elevated magnesium environment. Its robust mechanical properties, magnesium encapsulation capability, and biocompatibility underscore its potential as a valuable tool for advancing neurogenesis-based therapies.

### Magnesium ions promote the differentiation of ENCCs into neurons with electrophysiological activity in vivo

**Fig. 6:**
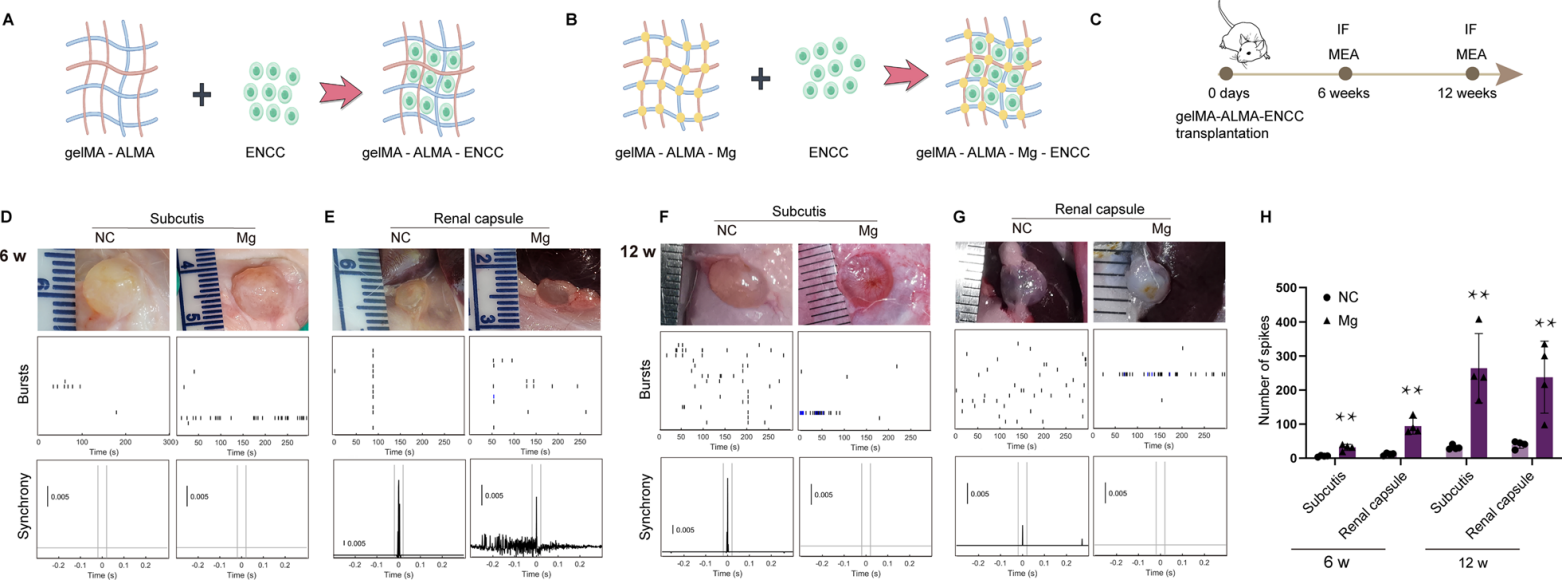
In vivo validation comparing the macroscopic appearance and electrical activity of magnesium-supplemented and non-supplemented ENCCs subcutaneous and renal ectopic transplantation at 6 and 12 weeks. **A**, **B** Schematic representation of gelMA and ALMA encapsulating ENCCs. **C** Timeline of the in vivo experimental procedure. **D**–**H** Both subcutaneous and renal capsule transplants of ENCCs at 6 and 12 weeks survive, with noticeable blood vessel surroundings for ENCCs colloid growth. MEA detection shows increased electrical activity in magnesium-supplemented ENCCs colloid in both subcutaneous and renal ectopic locations compared to the NC group (n = 4). NC: negative control, Mg: Mg.^2+^. Data were presented as mean ± SEM. *p < 0.05; **p < 0.01

**Fig. 7:**
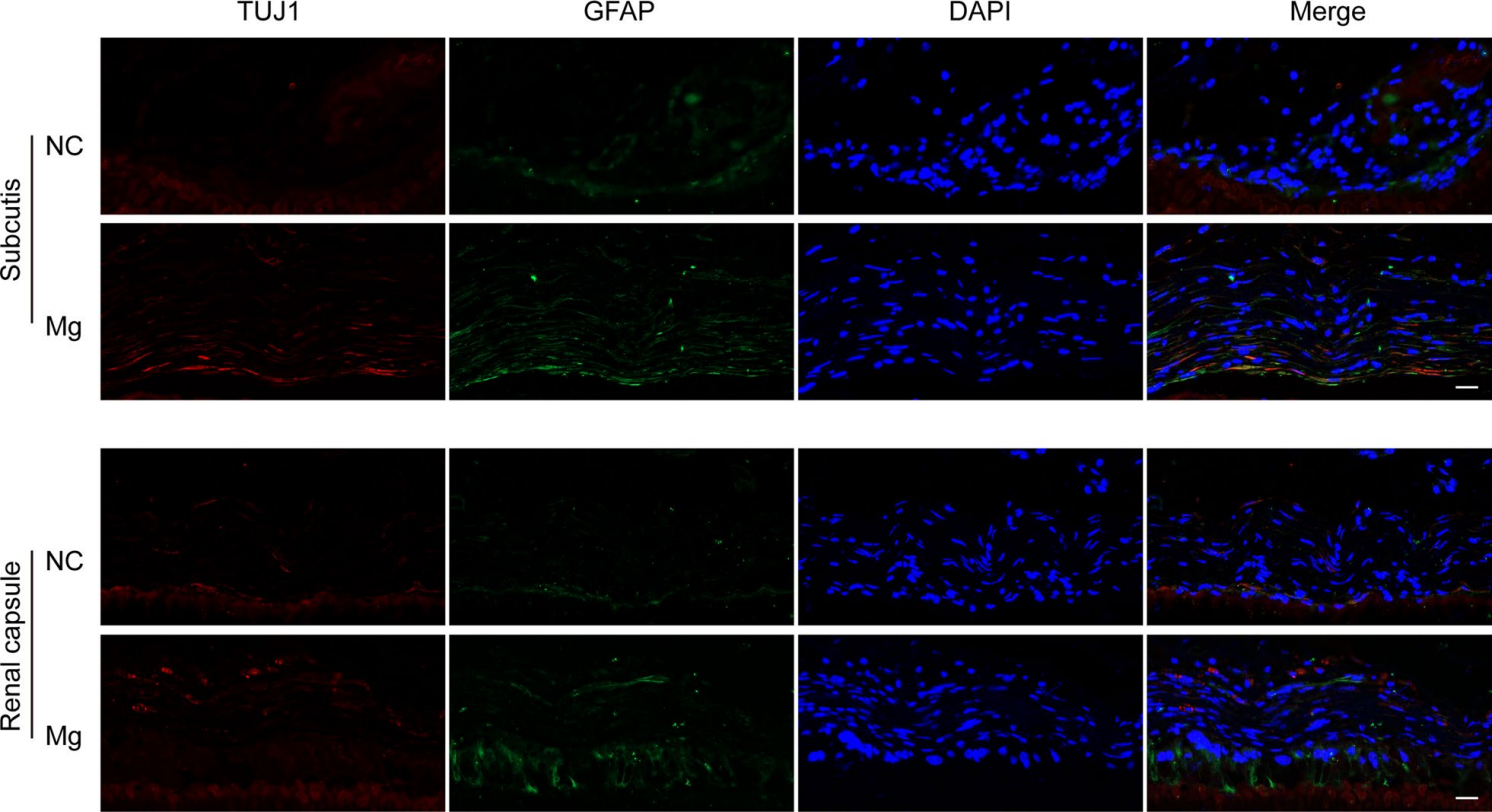
In vivo validation comparing the differentiation of magnesium- supplemented and non-supplemented ENCCs subcutaneous and renal ectopic transplants at 6 and 12 weeks. In both subcutaneous and renal ectopic sites, elevated expression of neuronal marker TUJ1 and neural glial cell marker GFAP is observed in the high magnesium environment, indicating enhanced neurogenesis (n = 5). NC: negative control, Mg: Mg^2+^. Scale bars: 20 μm

To further explore the impact of Mg^2+^ on the differentiation of ENCCs into neuronal cells in vivo, ENCCs encapsulated in gelMA-ALMA hydrogels with and without magnesium were transplanted subcutaneously and into renal capsules of SD rats. The encapsulation process and surgical timeline are illustrated in Fig. 6A–C. Post-transplantation, blood magnesium concentration tests revealed no increase in systemic magnesium levels (Figure Supplementary 5), indicating that gelMA-ALMA-Mg could locally release Mg^2+^ without entering the systemic circulation, thereby avoiding hypermagnesemia. At 6 and 12 weeks post-transplantation, the grafts were observed under the skin and in renal capsules, surrounded by vascularization, MEA testing indicated active potentials in grafts from both subcutaneous and renal capsule sites, with higher electrical activity frequency in the Mg group compared to the NC group (Fig. 6D–H). Immunofluorescence revealed elevated TUJ1 expression in the gelMA-ALMA-Mg group compared to the gelMA-ALMA group at both 6 and 12 weeks, suggesting that Mg^2+^ promote the differentiation of ENCCs into neuronal cells in vivo (Fig. 7).

## Discussion

The study unveils the significant impact of Mg^2+^ on the differentiation of ENCCs into neuronal cells. Within the concentration range of 1–100 mM, Mg^2+^ enhance ENCCs viability, with 10 mM identified as the optimal concentration for promoting differentiation. Besides, Mg^2+^ not only enhances ENCCs viability but also mitigates ROS damage and increases NAD + generation. This elevation in magnesium levels results in increased expression of neural markers (TUJ1 and UCHL1) and functional neuronal characteristics in ENCCs. Seahorse analysis indicates that Mg^2+^ primarily influence cellular metabolism through the glycolytic pathway during ENCCs differentiation, with no notable alterations in the TCA pathway. Inhibiting glycolysis with 2-DG suppresses ENCCs differentiation, emphasizing the importance of glycolysis in this process. Untargeted metabolomic profiling suggests an enhancement in the metabolic capacity of ENCCs under the influence of Mg^2+^. The gelMA-ALMA hydrogel, serving as a magnesium reservoir, exhibits commendable mechanical properties and biocompatibility, facilitating ENCCs differentiation into neurons in vivo (Graphical abstract).

In recent years, the Warburg effect, characterized by enhanced glycolysis, has been recognized as a metabolic hallmark of cancer cells [21], and it has also garnered widespread attention in other diseases. The neural crest is a migratory cell population in the embryo of vertebrates that undergoes extensive metabolic remodeling to participate in aerobic glycolysis. Increased glycolytic flux promotes Yap/Tead signaling, activating the expression of a series of transcription factors that drive the conversion of epithelial cells to mesenchymal cells [10]. In this study, we propose an alternative view, demonstrating that Mg^2+^ can induce a Warburg effect-like response by increasing glycolytic flux to promote the differentiation of ENCCs into neurons. Glycolysis stress experiments show that Mg^2+^ mainly influences cellular metabolism through the glycolytic pathway, and importantly, there are no significant changes in the TCA pathway (Figs. 2, 3). This is consistent with previous studies, emphasizing the role of glycolysis in cellular differentiation, especially in the context of neurogenesis [22]. Inhibiting glycolysis with 2-DG and 3-BP significantly reduces lactate production and suppresses ENCCs differentiation. This underscores the importance of glycolysis in the differentiation process, a phenomenon observed in other cell types as well [23–26]. Under high magnesium conditions, the increase in key glycolytic enzymes (GLUT1, HK2, PFKFB3, and LDHA) further confirms the reliance of ENCCs on glycolysis for energy production during differentiation. Reverse validation using 2-DG and 3-BP adds robustness to these findings. Untargeted metabolomic analysis shows an overall enhancement in the metabolic capacity of ENCCs under the influence of Mg^2+^, with 59 metabolites upregulated, mainly belonging to various amino acids. This suggests that upregulated amino acids may play a crucial role in supporting the increased energy demand associated with neurogenesis (27, 28). However, further research is needed to elucidate the specific metabolic pathways affected by these amino acids. It is important to note that in this study, the induction period was extended to 14 and 28 days because preliminary experiments using MEA failed to detect electrical signals from neurons derived from ENCCs after 7 days (Fig. 1D).

The gastrointestinal tract is controlled by a complex neural network that coordinates essential physiological functions. Promoting the differentiation of ENCCs into neurons has long been a focus of scientific inquiry and a key area of research in the transplantation of stem cells for intestinal neurodevelopmental disorders. Recent studies have found that GDNF enemas can promote the regeneration of functional ENS cells in situ. GDNF administration has also been shown to prevent mortality in different gene-specific HSCR mouse models, inducing neurogenesis in the intestine and improving colonic structure and function. Exogenous GDNF can penetrate the permeable distal colon, leading to increased endogenous GDNF and RET levels throughout the colon [29].

The role of magnesium in neurogenesis has been increasingly recognized. Elevated levels of magnesium in the brain have been shown to enhance memory and synaptic plasticity [30]. Moreover, increased magnesium levels promote neuronal cell differentiation by activating the ERK/CREB pathway while inhibiting glial differentiation of adult mouse neural progenitor cells in primary culture [31].

The utilization of the gelMA-ALMA hydrogel as a local magnesium reservoir presented a promising strategy to enhance ENCCs differentiation in vivo. The hydrogel's dual-crosslinked nature, combining photo cross-linkable gelMA and ALMA, showcased excellent mechanical properties and biocompatibility [32–35]. The observed lower swelling rates and superior, uniform pore structure of gelMA-ALMA compared to individual gelMA and ALMA underline its potential as an effective scaffold for cell encapsulation. In vivo transplantation of ENCCs encapsulated in gelMA-ALMA hydrogels, with and without magnesium, revealed intriguing results. Blood magnesium concentration tests indicated that gelMA-ALMA-Mg could locally release Mg^2+^ without causing systemic hypermagnesemia. The presence of structurally and functionally intact intestinal tissues, along with elevated TUJ1 expression in the gelMA-ALMA-Mg group, signifies the positive impact of Mg^2+^ on ENCCs differentiation in vivo. These findings align with recent studies showcasing the potential of hydrogels in tissue engineering and regenerative medicine [36–39]. To better adapt to the anatomical and physiological structure of the gastrointestinal tract, in subsequent studies, we will develop magnesium-loaded three-dimensional scaffolds more suitable for ENCCs growth. These will be fabricated into a film using electrospinning for colonic in situ transplantation, thereby avoiding intestinal wall swelling and narrowing due to excessive graft volume.

## Conclusions

This study suggests that magnesium ions can significantly promote the differentiation of ENCCs into neuronal cells, primarily by activating the Warburg effect. Additionally, the gelMA-ALMA hydrogel, serving as a local magnesium reservoir, exhibits the potential to enhance ENCCs differentiation into neurons in vivo.

## Supplementary Information

Supplementary Material. Figure 1. Flow cytometry identification of ENCCs with over 90% positivity for P75-APC

Supplementary Material. Figure 2. In vitro assessment of the impact of different magnesium ion concentrations on ENCCs proliferation and apoptosis.Gradual concentration treatment of ENCCs for 48 hours reveals the lowest total apoptosis rateat 10mM magnesium ion concentration.CCK-8 results show the highest OD value at 10mM magnesium ion concentration, indicating maximal ENCCs viability. NC: negative control. Data were presented as mean ± SEM. *p < 0.05; **p < 0.01.

Supplementary Material. Figure 3. Enhanced metabolism of ENCCs differentiation into neurons under high magnesium conditions. Untargeted metabolomics identifies 59 upregulated metabolites and 11 downregulated metabolites. NC: negative control, Mg: Mg^2+^.

Supplementary Material. Figure 4. Evaluation of gelMA and ALMA performance.Schematic representation of the gelMA and ALMA hydrogel scaffold materials.Macroscopic view and scanning electron microscopy images of gelMA, ALMA, and gelMA+ALMA.Elastic modulus of gelMA+ALMA is higher than using gelMA or ALMA alone.Swelling ratio of gelMA+ALMA is lower than using gelMA or ALMA alone. NC: negative control, Mg: Mg^2+^. Data were presented as mean ± SEM. * Comparison between ALMA+gelMA and ALMA, # Comparison between ALMA+gelMA and gelMA, ## and **p < 0.01.

Supplementary Material. Figure 5. After ectopic transplantation of ENCCs, blood magnesium and calcium changes were assessed on post-transplantation days 0, 1, 4, and 7 using inductively coupled plasma mass spectrometry. Mg: Mg^2+^, Ca: calcium ion. Data were presented as mean ± SEM.

Supplementary Material. Figure 6.

Supplementary Material 7.

Supplementary Material 8.

Supplementary Material 9.

Supplementary Material 10.

Supplementary Material 11.

Supplementary Material 12.

Supplementary Material 13.

Supplementary Material 14.

Supplementary Material 15.

Supplementary Material 16. The uncropped gel electrophoresis image for Figure 1A, 2C, 4B and 5A

Supplementary Material 17.

## Data Availability

The mass spectroscopy data (isotopomers measurement and metabolomic sequencing) supporting the results reported are archived in supplementary files. The datasets generated during and analysed during the current study are available from the corresponding author on reasonable request.
