# Supplementary figures and images for: Magnesium ions regulate the Warburg effect to promote the differentiation of enteric neural crest cells into neurons
Source: Stem Cell Res Ther. 2025 Jan 23;16:19. doi: 10.1186/s13287-024-04121-4 (PMC11755793; doi:10.1186/s13287-024-04121-4)

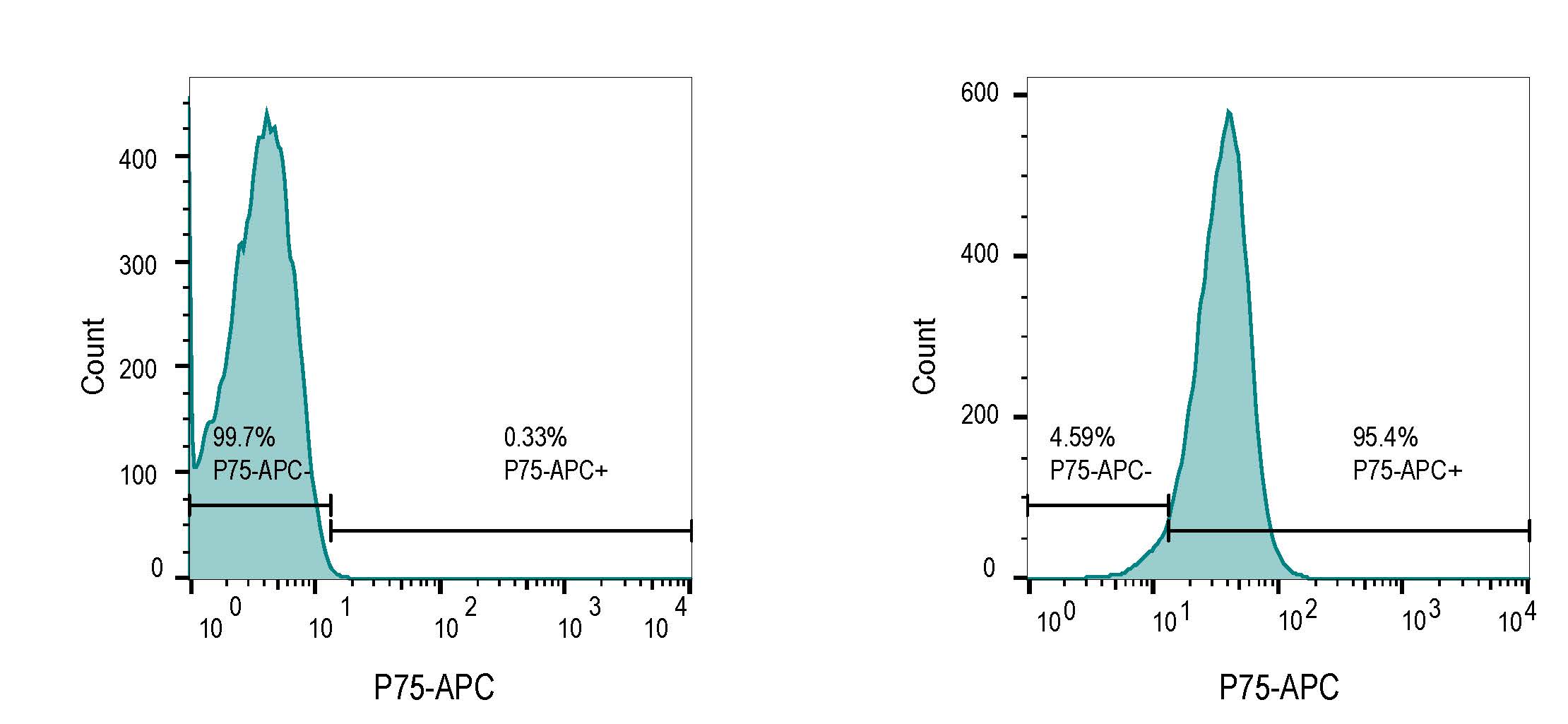

Supplement: Supplementary file 1 — Supplementary Material. Figure 1. Flow cytometry identification of ENCCs with over 90% positivity for P75-APC [file 13287_2024_4121_MOESM1_ESM.jpg]

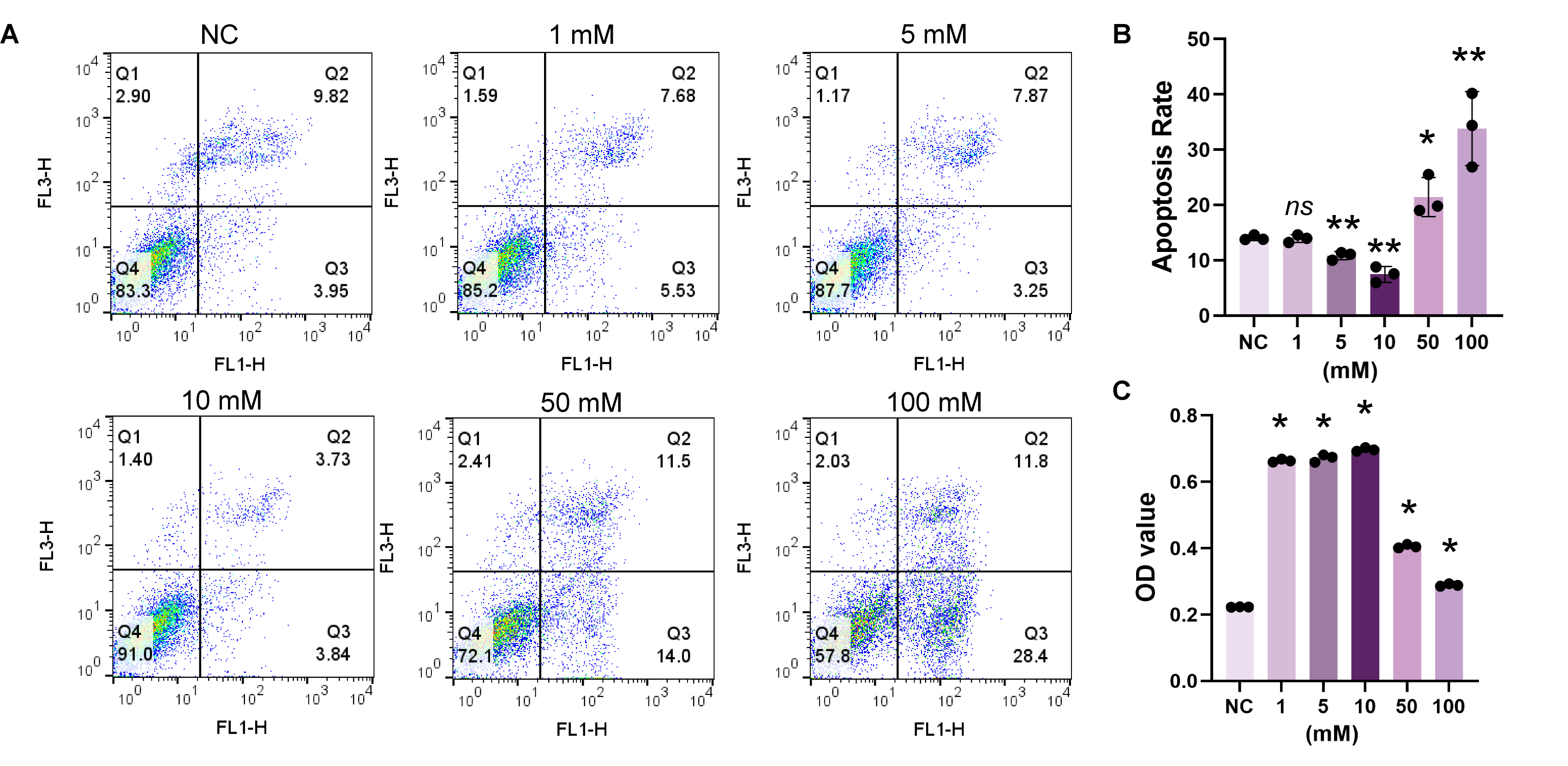

Supplement: Supplementary file 2 — Supplementary Material. Figure 2. In vitro assessment of the impact of different magnesium ion concentrations on ENCCs proliferation and apoptosis.Gradual concentration treatment of ENCCs for 48 hours reveals the lowest total apoptosis rateat 10mM magnesium ion concentration.CCK-8 results show the highest OD value at 10mM magnesium ion concentration, indicating maximal ENCCs viability. NC: negative control. Data were presented as mean ± SEM. *p < 0.05; **p < 0.01. [file 13287_2024_4121_MOESM2_ESM.tif]

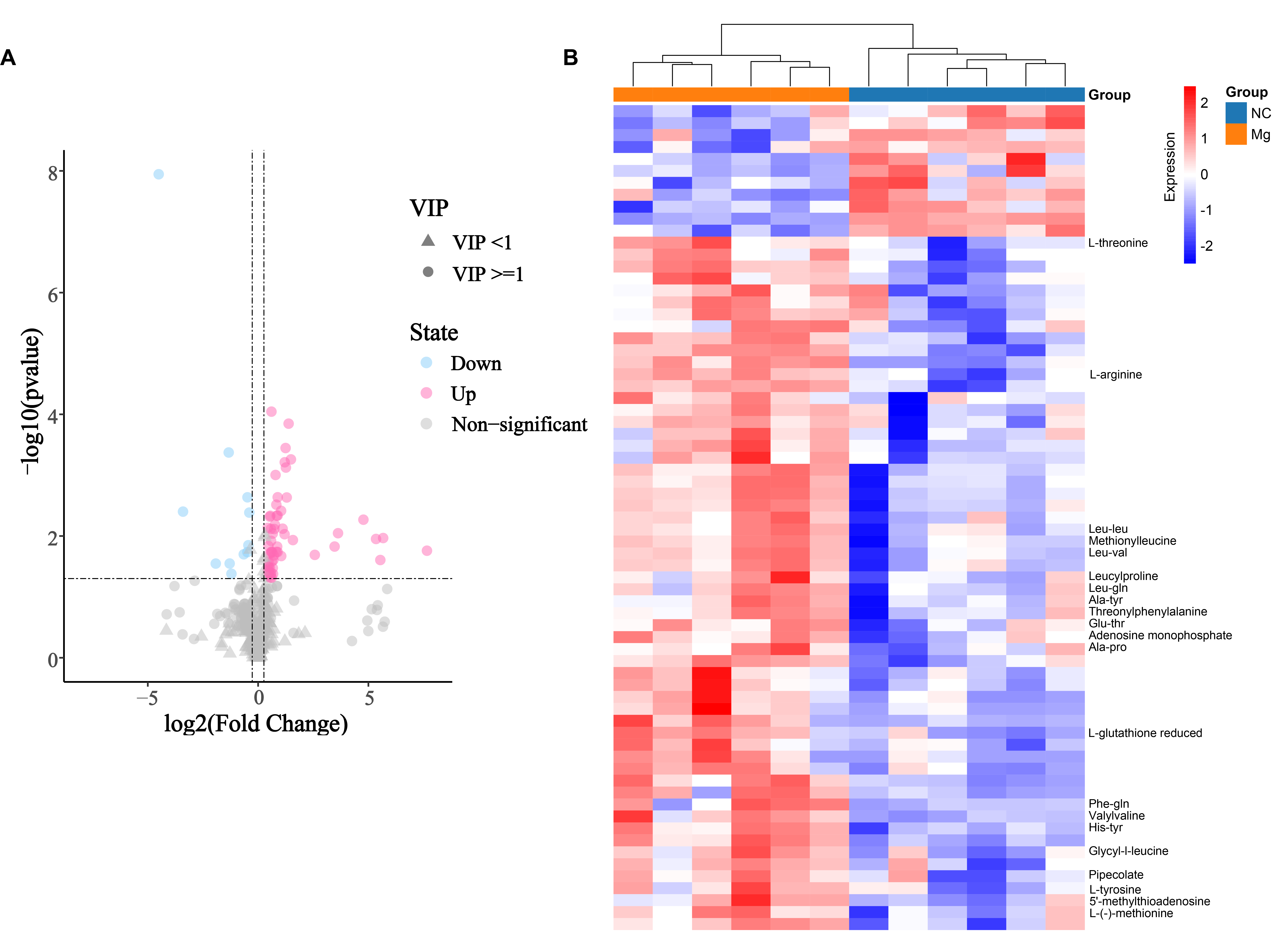

Supplement: Supplementary file 3 — Supplementary Material. Figure 3. Enhanced metabolism of ENCCs differentiation into neurons under high magnesium conditions. Untargeted metabolomics identifies 59 upregulated metabolites and 11 downregulated metabolites. NC: negative control, Mg: Mg2+. [file 13287_2024_4121_MOESM3_ESM.png]

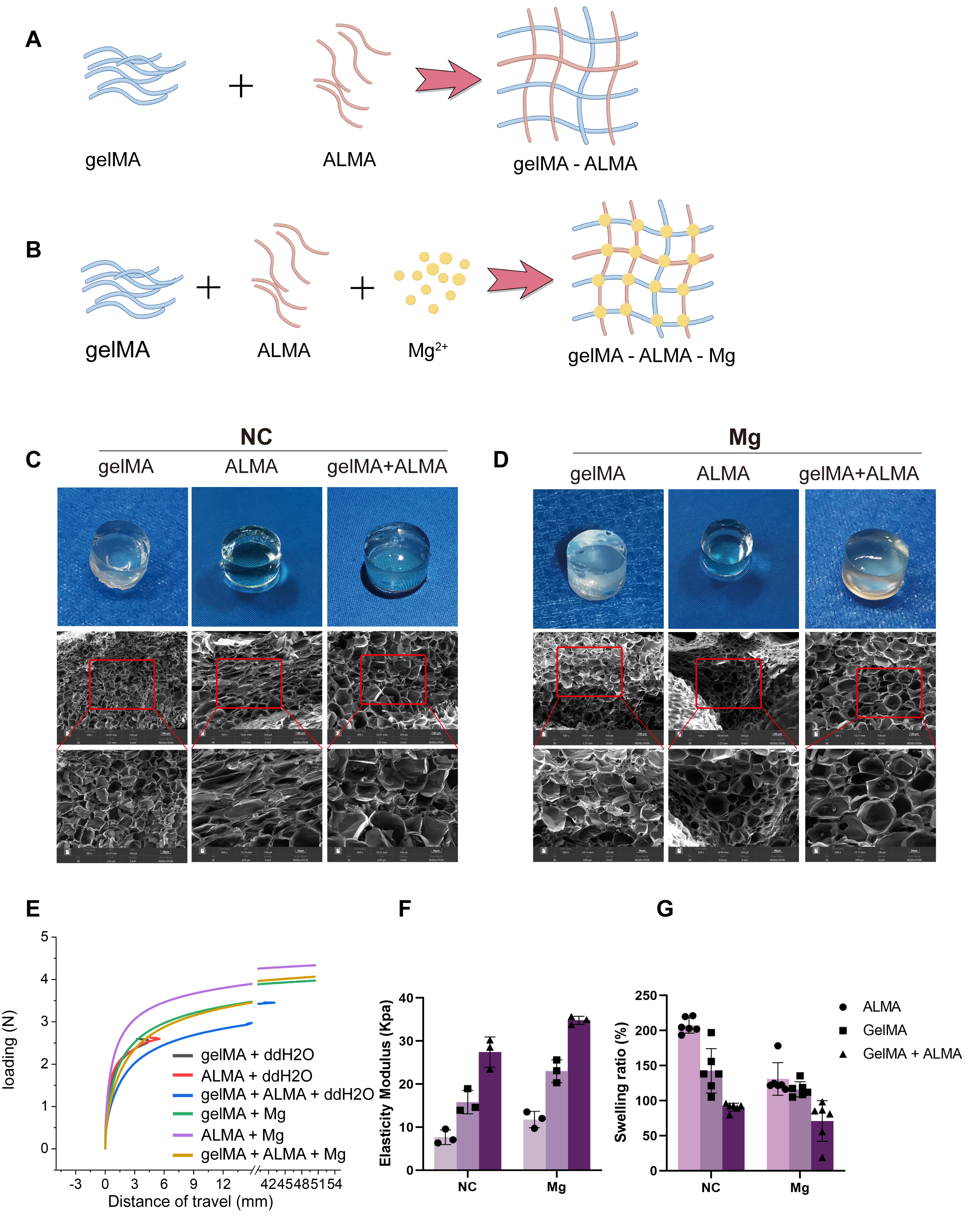

Supplement: Supplementary file 4 — Supplementary Material. Figure 4. Evaluation of gelMA and ALMA performance.Schematic representation of the gelMA and ALMA hydrogel scaffold materials.Macroscopic view and scanning electron microscopy images of gelMA, ALMA, and gelMA+ALMA.Elastic modulus of gelMA+ALMA is higher than using gelMA or ALMA alone.Swelling ratio of gelMA+ALMA is lower than using gelMA or ALMA alone. NC: negative control, Mg: Mg2+. Data were presented as mean ± SEM. * Comparison between ALMA+gelMA and ALMA, # Comparison between ALMA+gelMA and gelMA, ## and **p < 0.01. [file 13287_2024_4121_MOESM4_ESM.tif]

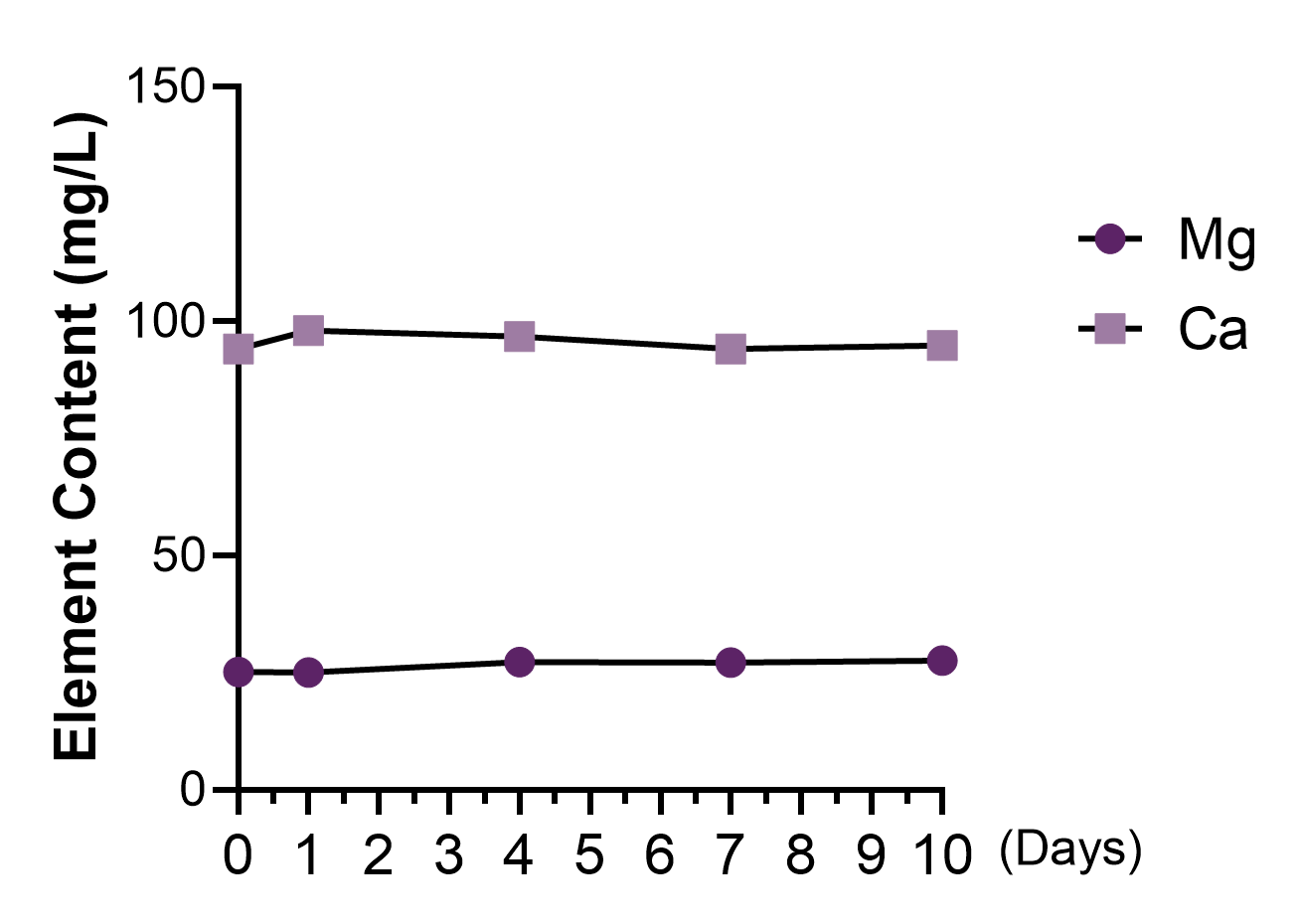

Supplement: Supplementary file 5 — Supplementary Material. Figure 5. After ectopic transplantation of ENCCs, blood magnesium and calcium changes were assessed on post-transplantation days 0, 1, 4, and 7 using inductively coupled plasma mass spectrometry. Mg: Mg2+, Ca: calcium ion. Data were presented as mean ± SEM. [file 13287_2024_4121_MOESM5_ESM.png]

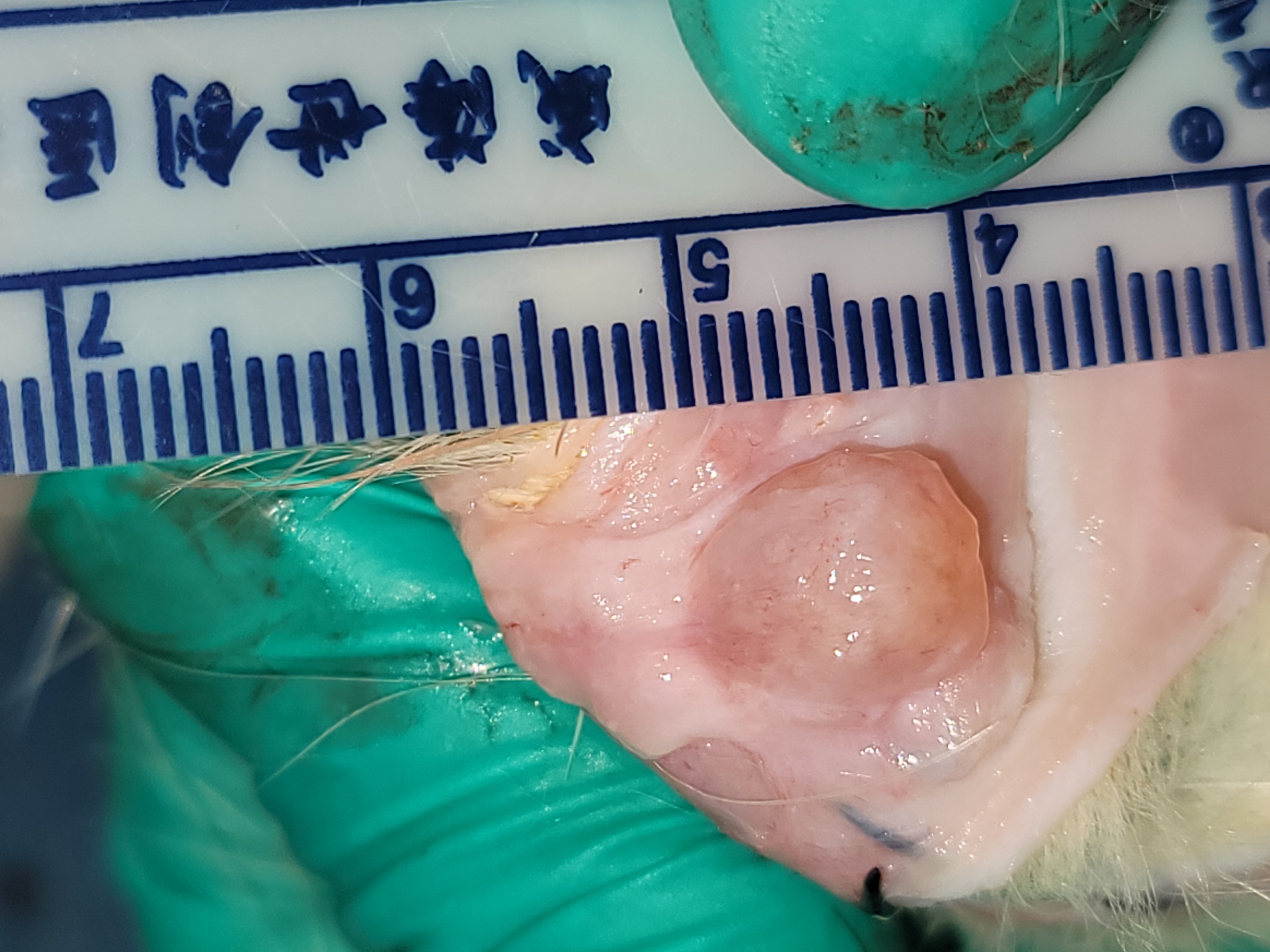

Supplement: Supplementary file 6 — Supplementary Material. Figure 6. [file 13287_2024_4121_MOESM6_ESM.jpg]

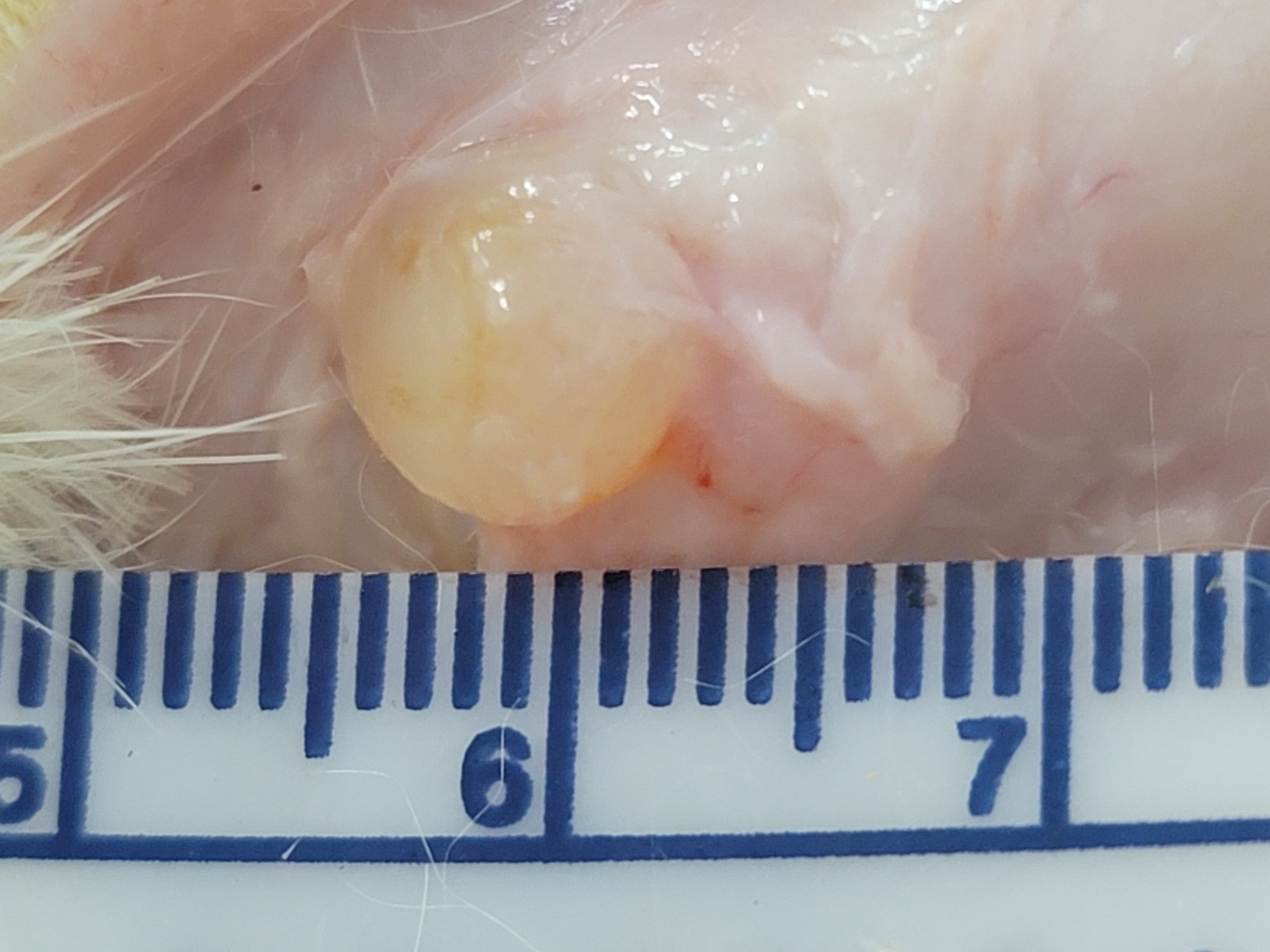

Supplement: Supplementary file 7 — Supplementary Material 7. [file 13287_2024_4121_MOESM7_ESM.jpg]

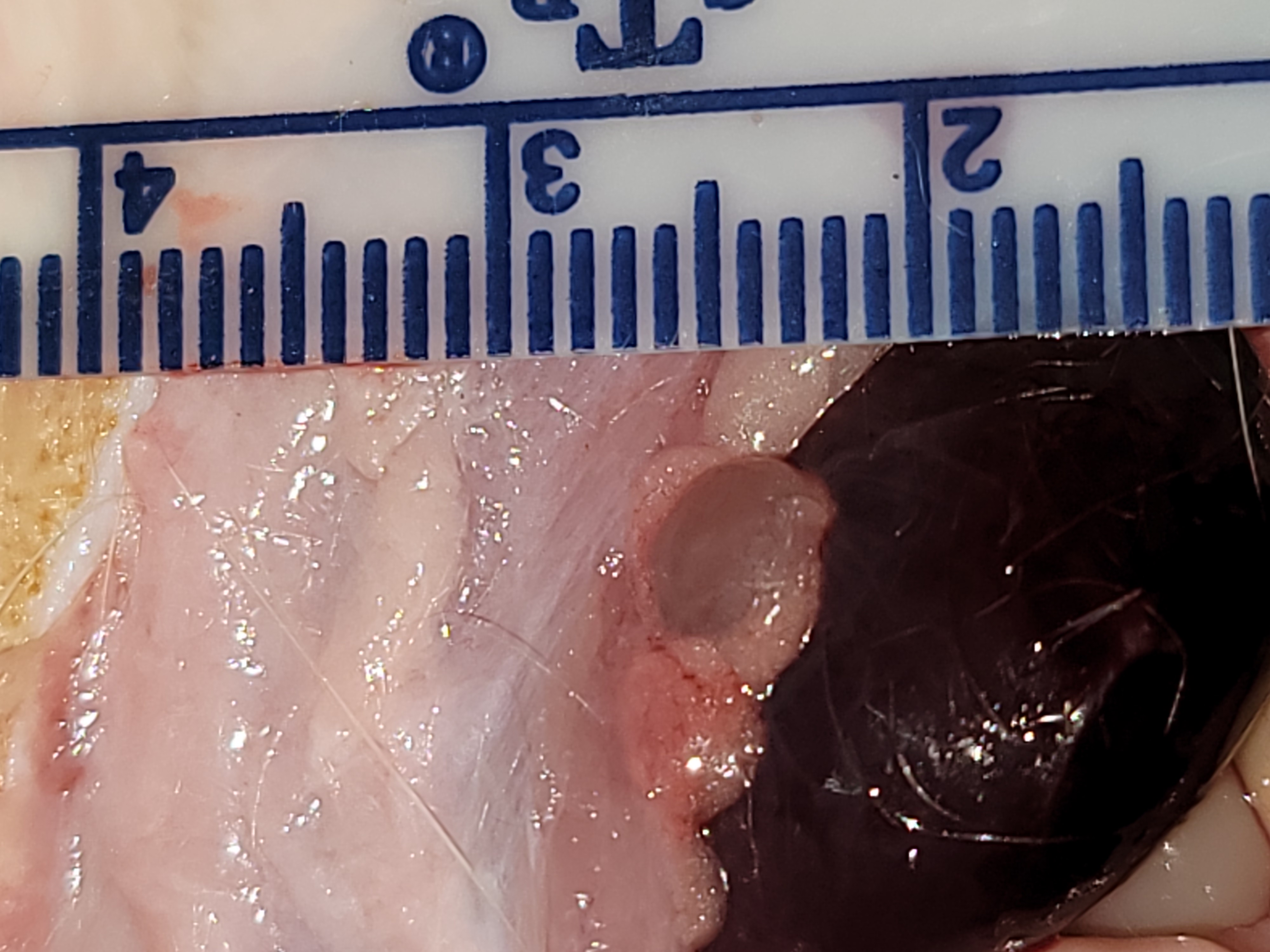

Supplement: Supplementary file 8 — Supplementary Material 8. [file 13287_2024_4121_MOESM8_ESM.jpg]

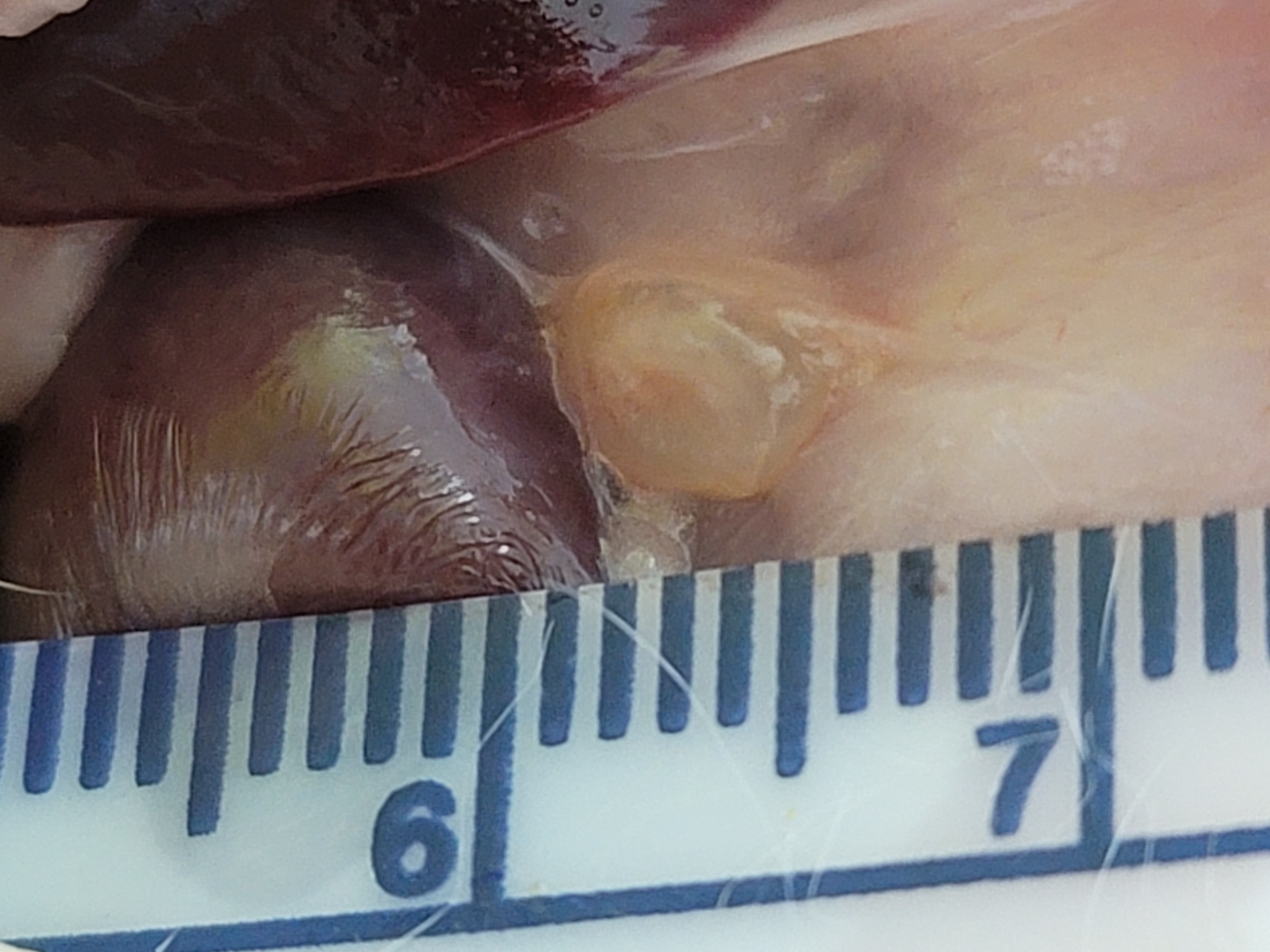

Supplement: Supplementary file 9 — Supplementary Material 9. [file 13287_2024_4121_MOESM9_ESM.jpg]

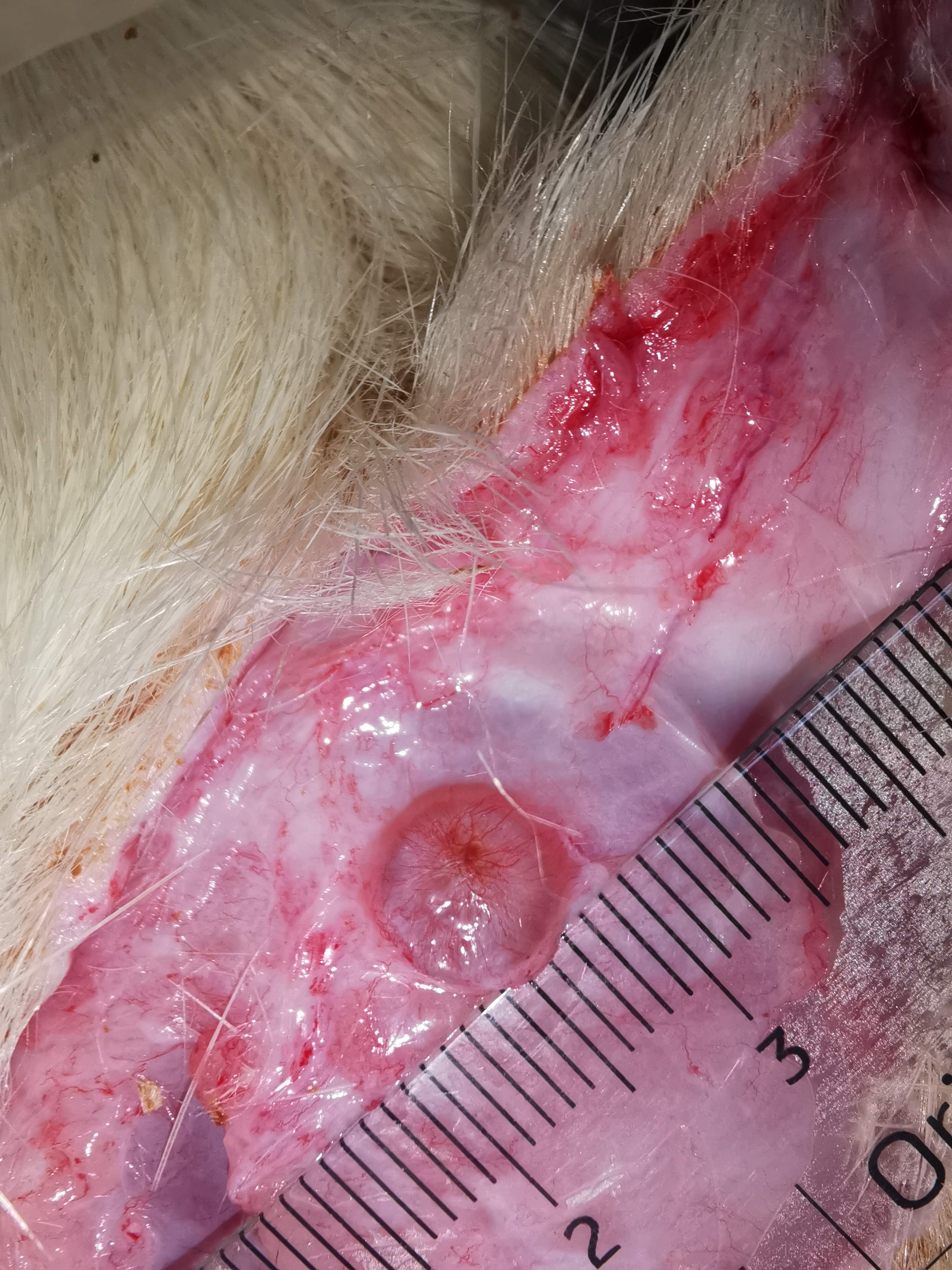

Supplement: Supplementary file 10 — Supplementary Material 10. [file 13287_2024_4121_MOESM10_ESM.jpg]

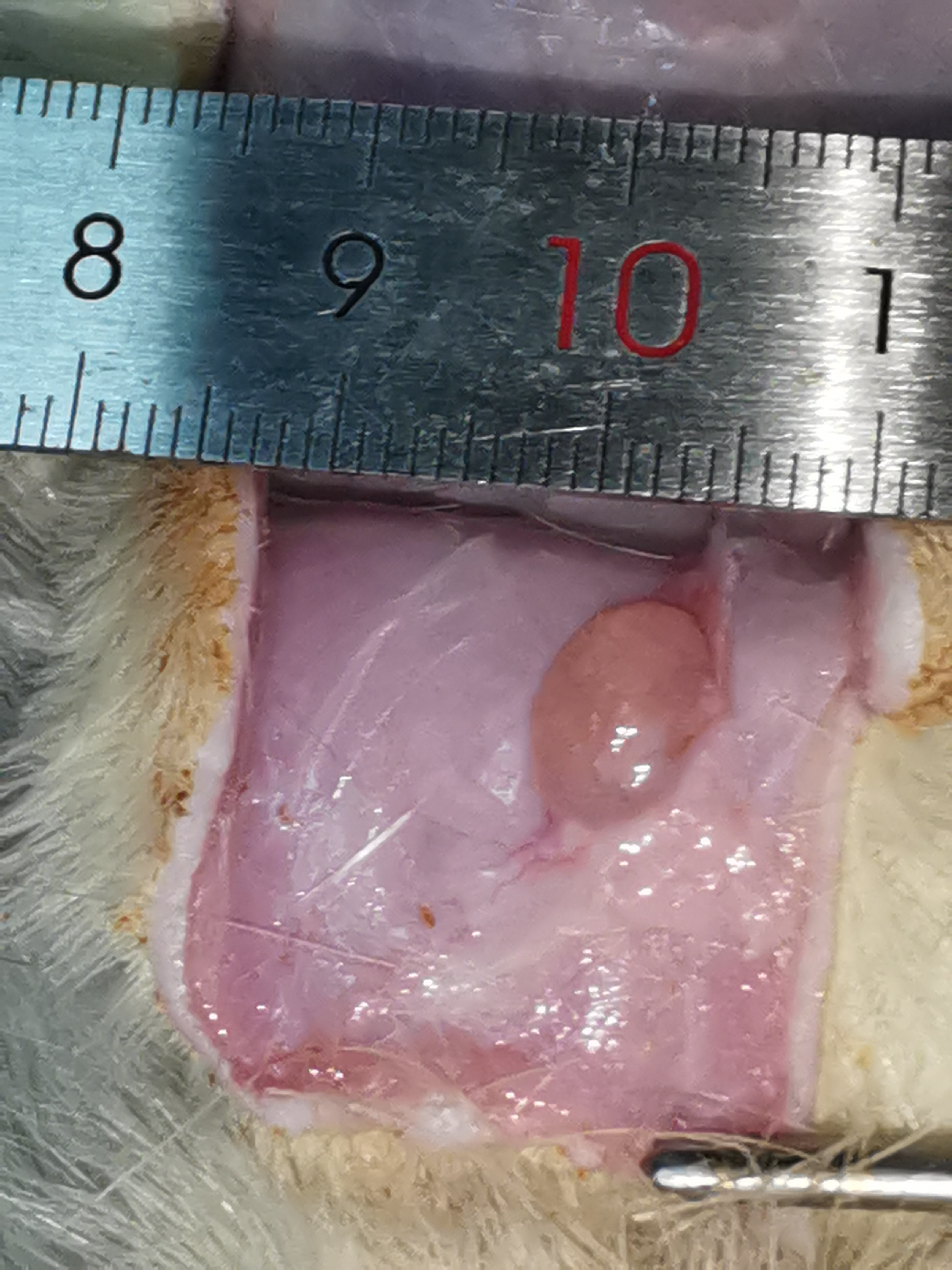

Supplement: Supplementary file 11 — Supplementary Material 11. [file 13287_2024_4121_MOESM11_ESM.jpg]

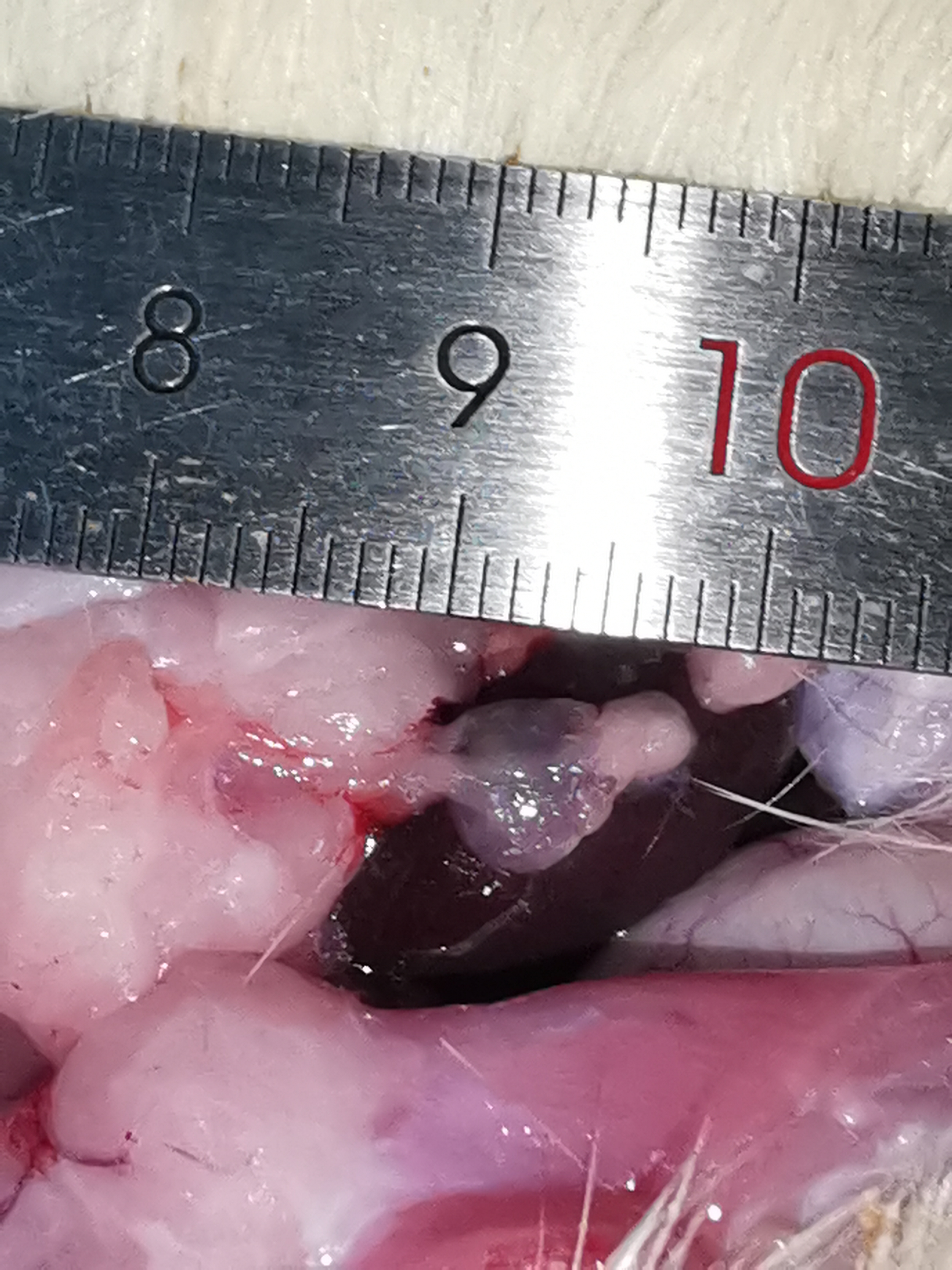

Supplement: Supplementary file 12 — Supplementary Material 12. [file 13287_2024_4121_MOESM12_ESM.jpg]

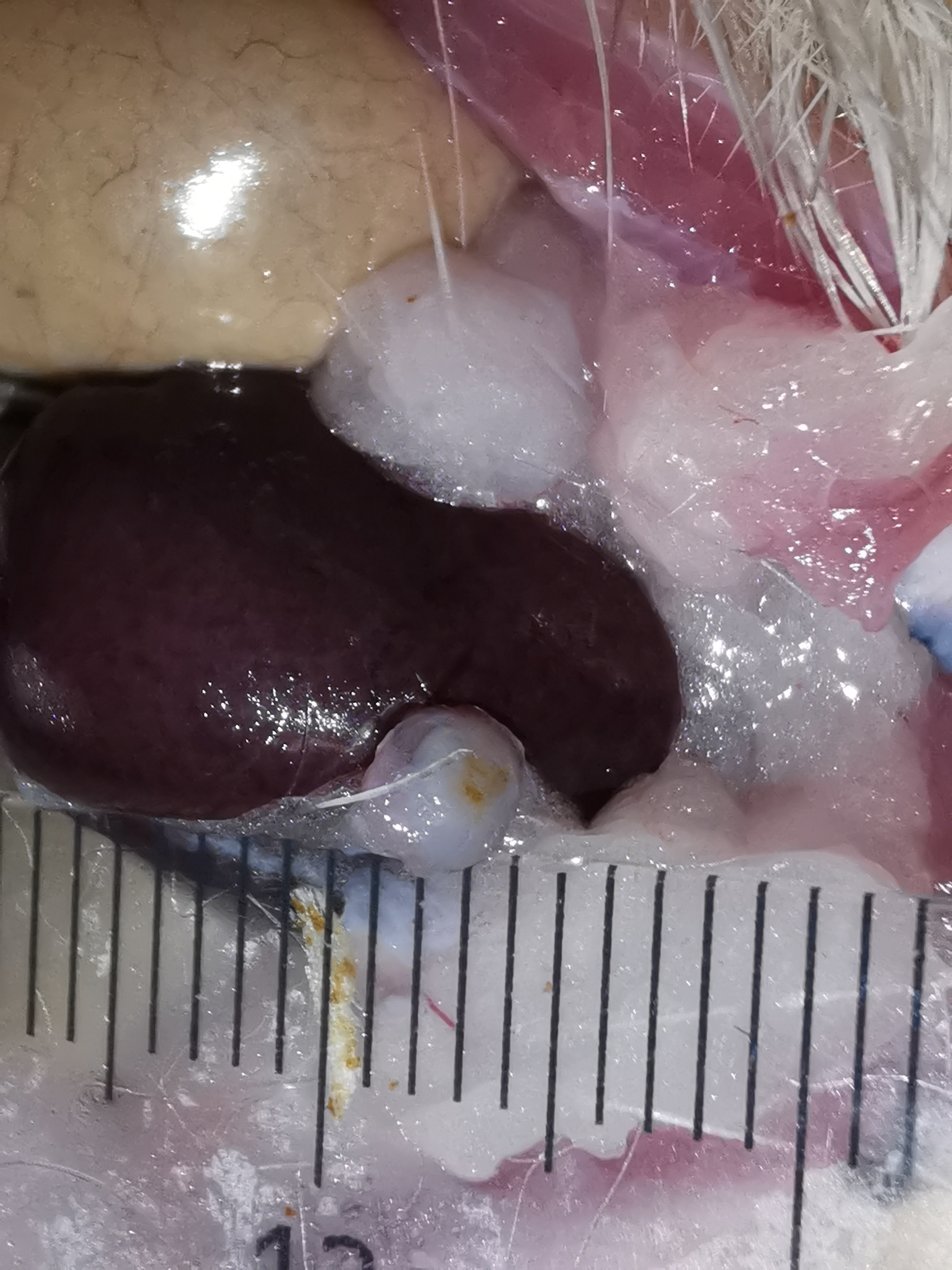

Supplement: Supplementary file 13 — Supplementary Material 13. [file 13287_2024_4121_MOESM13_ESM.jpg]

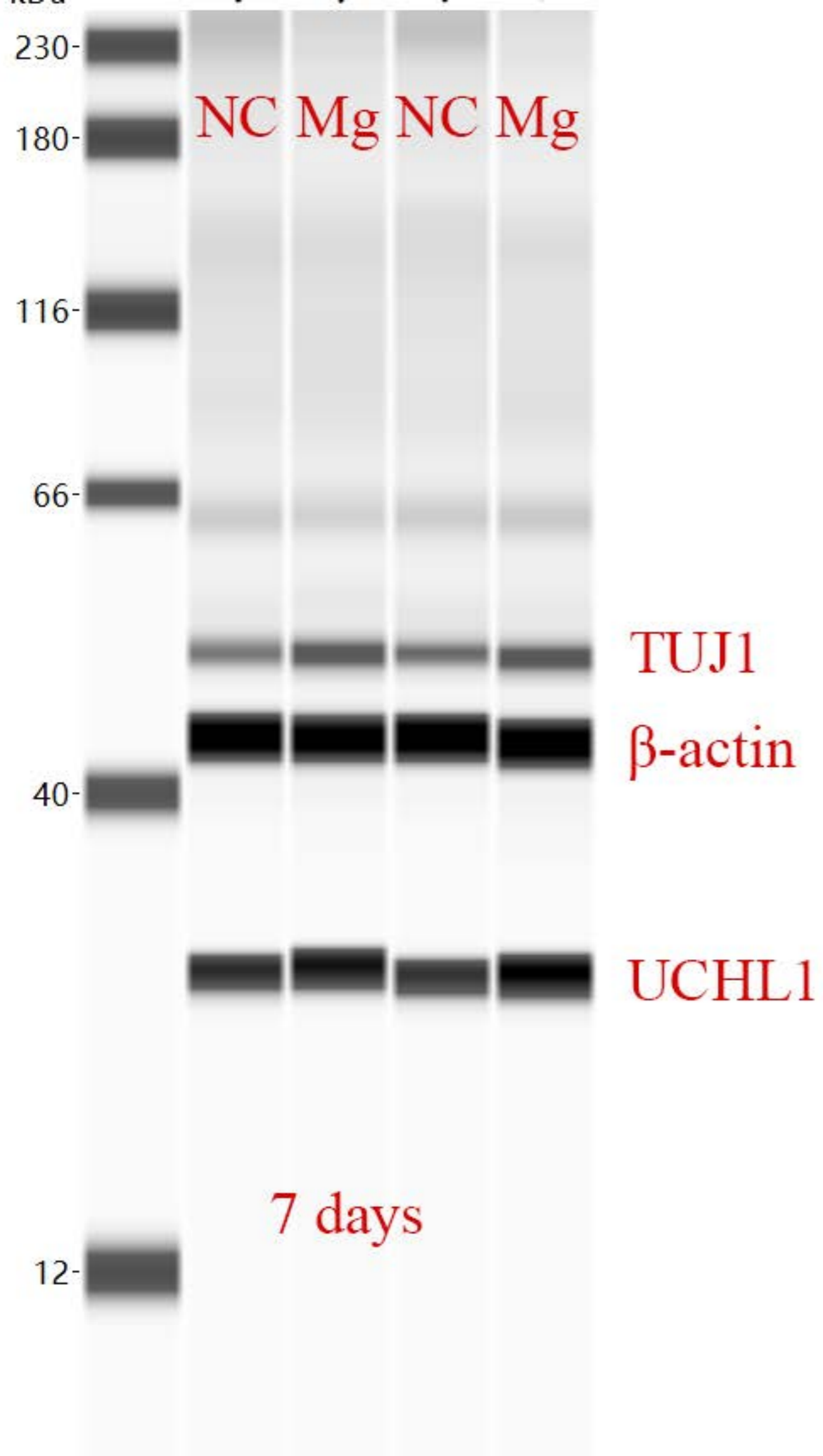

**B**

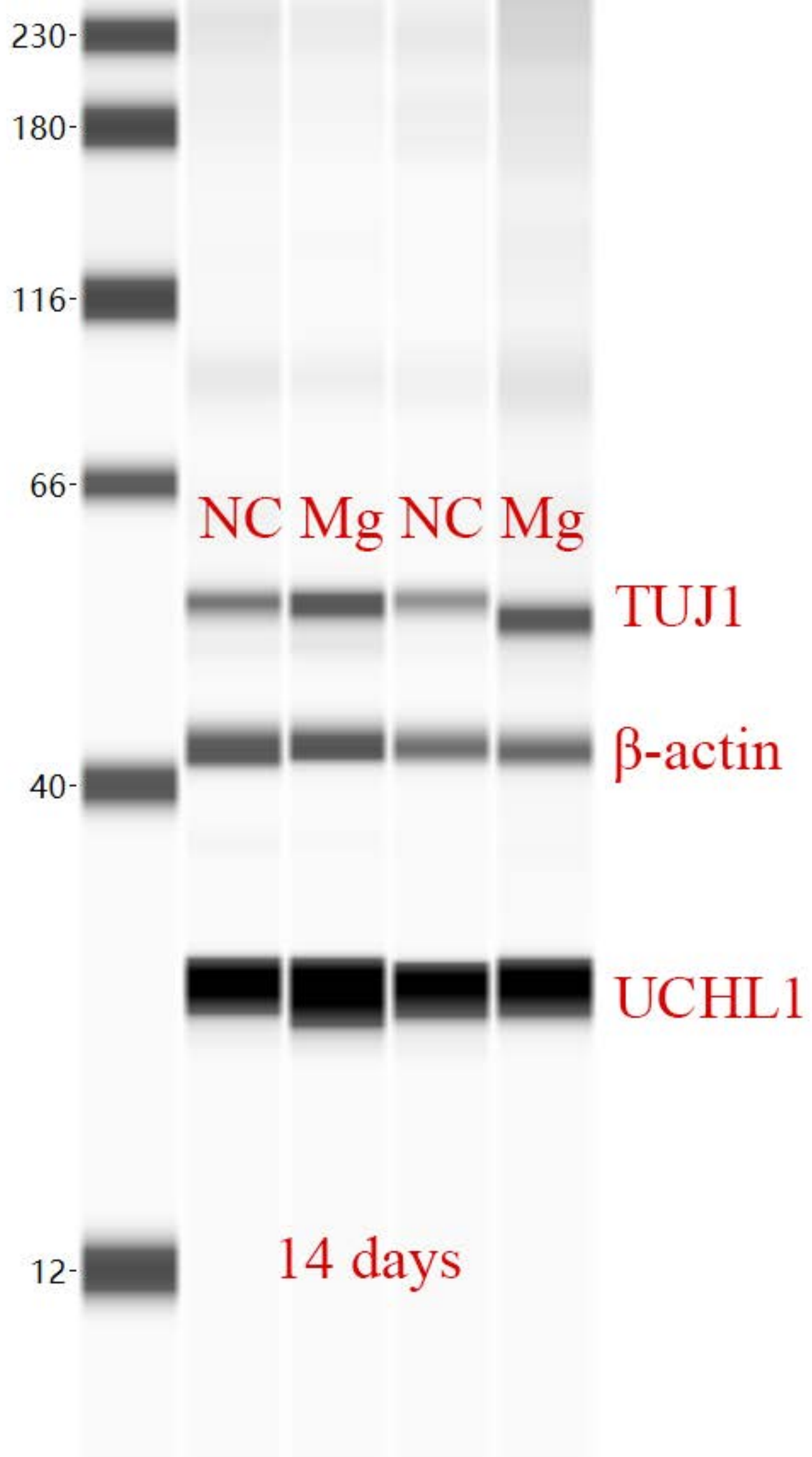

C

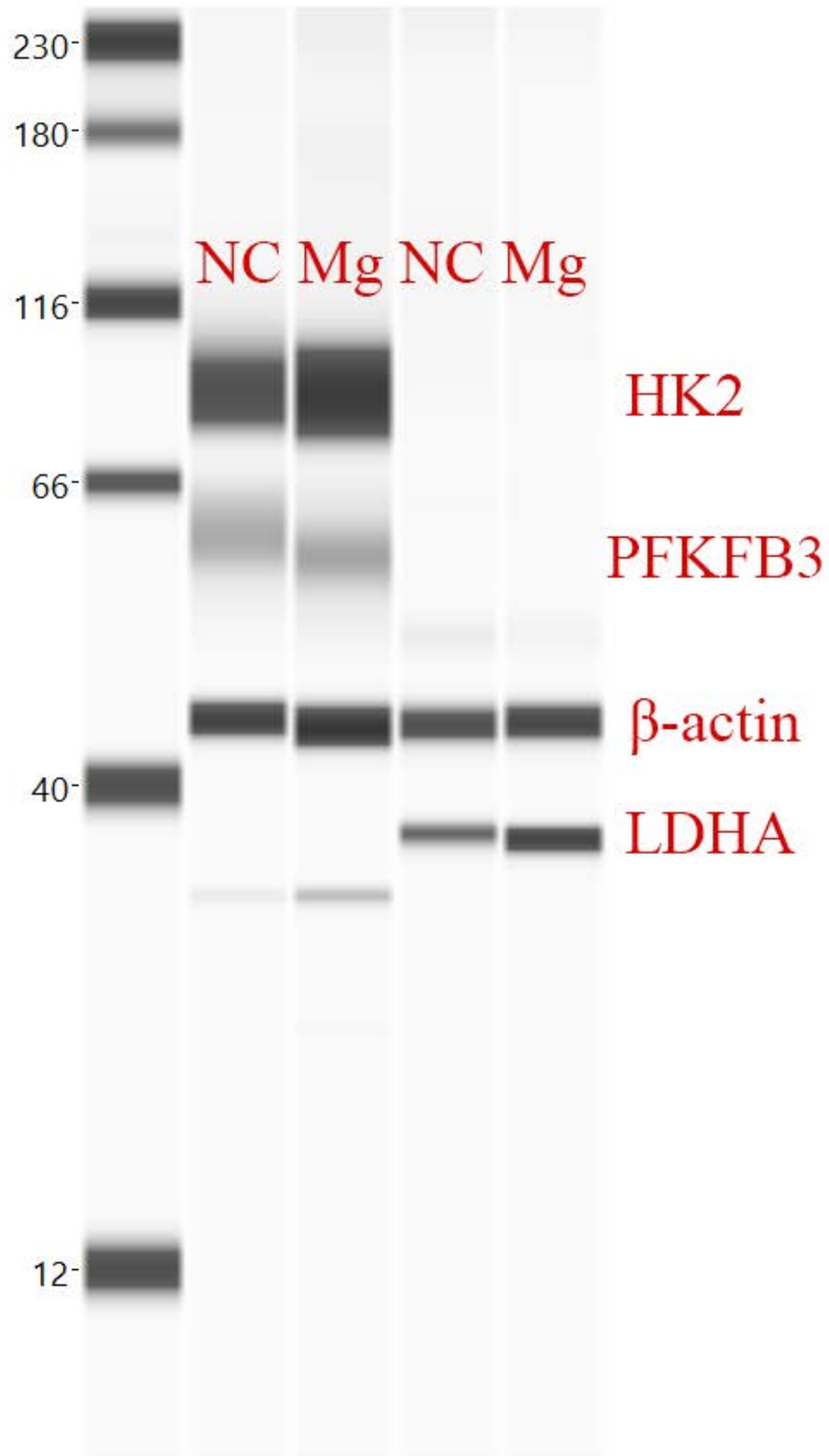

D

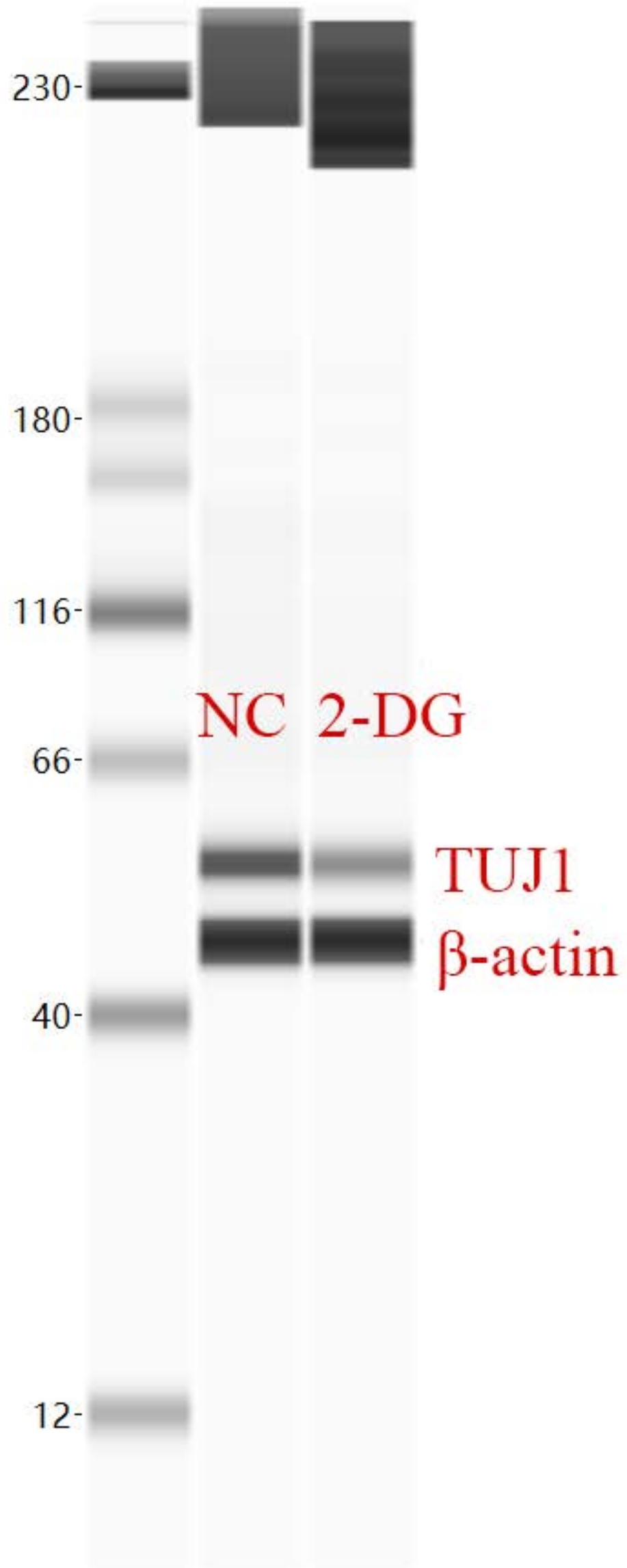

**E**

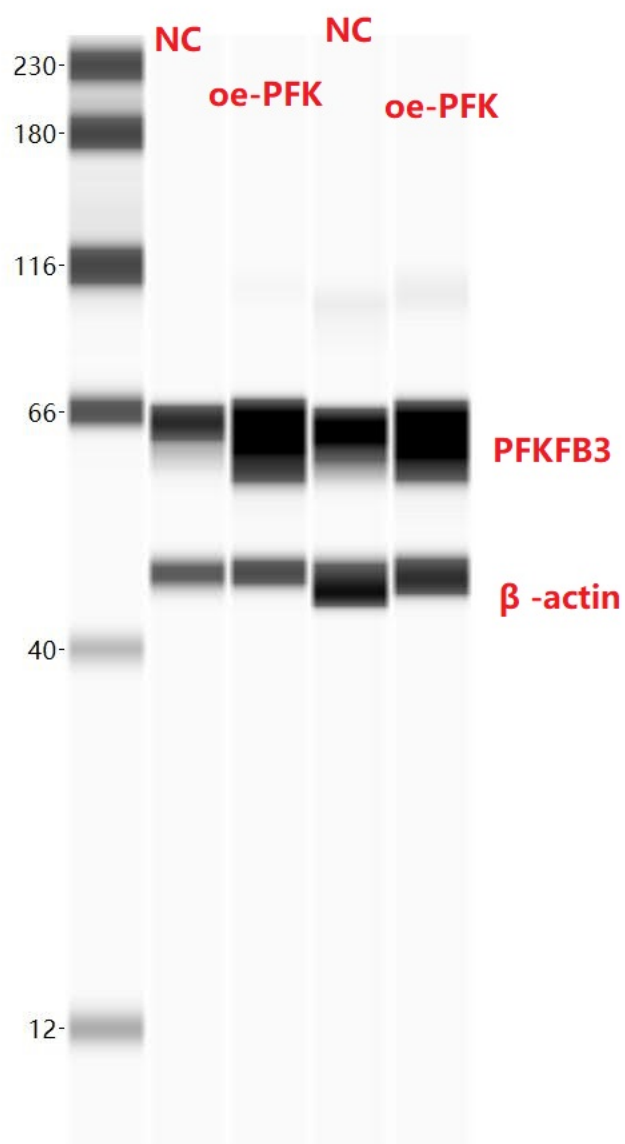

Supplement: Supplementary file 16 — Supplementary Material 16. The uncropped gel electrophoresis image for Figure 1A, 2C, 4B and 5A [file 13287_2024_4121_MOESM16_ESM.pdf]
