# Supplementary material for: Magnesium ions regulate the Warburg effect to promote the differentiation of enteric neural crest cells into neurons
Source: Stem Cell Res Ther. 2025 Jan 23;16:19. doi: 10.1186/s13287-024-04121-4 (PMC11755793; doi:10.1186/s13287-024-04121-4)
Supplement: Supplementary file 17 — Supplementary Material 17. [file 13287_2024_4121_MOESM17_ESM.docx]

**Magnesium Ions Regulate the Warburg Effect to Promote the** **Differentiation of** **Enteric Neural Crest Cells into** **Neurons**

**1 Material and Methods**

**1.1 The impact of magnesium ion on cells proliferation and apoptosis**

The influence of magnesium chloride (MgCl_2_) on cell proliferation and apoptosis was assessed using the CCK-8 method and flow cytometry. ENCCs were seeded in 96-well plates at a density of 5000 cells per well and exposed to different doses of MgCl_2_ for 48 hours. Subsequently, 100 μL of 10% CCK-8 solution (Cat. GK10001) was added to each well, followed by incubation for a specified time. Absorbance was measured at 450 nm using a microplate reader. After completing the experimental treatment, cells were collected, washed with pre-cooled PBS, and cell proliferation capacity was evaluated based on Optical Density (OD) values. After completing the experimental treatment, cells were suspended in 1× binding buffer at a concentration of 1 × 10^6^ cells/mL. Flow cytometry was performed using the cell apoptosis detection kit (556547, BD). The cell suspension was centrifuged at 1000 rpm for 5 minutes. Following the manufacturer's instructions, each sample was treated with 0.5 μL FITC-conjugated Annexin V and 5 μL propidium iodide at room temperature in the dark for 20 minutes. After washing twice with 1× binding buffer to remove unbound dye, all samples were analyzed using a BD FACS Calibur flow cytometer (BD Biosciences, NJ, USA).

**1.2 Quantitative real-time PCR (qRT-PCR)**

Total mRNA was extracted using RNAfast200 (Fastagen, China) and then converted into cDNA using reverse transcription reagent kit (TOYOBO, Cat FSQ-101), according to the manufacturer's instructions. To detect gene expression levels, a Kit (TAKARA, Cat RR820A) was used. β-actin mRNA served as the internal control as a housekeeping gene. The primer list for the target genes is presented in Table 1.

**1.3 Simple western (Jess by ProteinSimple)**

We employed Simple Western blot (WB) technology to investigate the mechanism by which Mg^2+^ promote ENCCs differentiation into neuronal cells and their impact on cell metabolism. Cells were cultured in 6-well plates for 24 hours until the confluence reached 70%. Subsequently, cells were exposed to neuronal differentiation medium for 7 and 14 days. Following different protocols, cells were lysed with RIPA buffer, and proteins were separated and quantified using the BCA assay. To ensure measurement accuracy, the final protein concentration for each sample was adjusted to 0.2 μg/μL. Protein samples were denatured at 95 °C for 5 minutes in 0.1x sample buffer and 5x master mix. Then, protein separation was achieved through capillary electrophoresis using the JESS system produced in the United States. Specific antibodies against TUJ1 (1:40, ab78078, Abcam), UCHL1 (1: 10000, 14730-1-AP, Protentech), HK2 (1:10000, 22029-1-AP, Protentech), PFKFB3 (1:1000, 13763-1-AP, Protentech) and LDHA (1:800, 19987-1-AP, Protentech) were diluted in antibody dilution buffer and added to the wells. Subsequently, 10 μL of streptavidin-HRP and secondary antibodies were added to the wells. The Compass software version SW 4.1.0 was used for quantitative analysis of protein expression levels, assessing the detected protein bands.

**1.4** **Immunofluorescence and immunohistochemistry**

Immunofluorescence was employed to detect the protein expression of target genes in cells. Cells were fixed with 4% paraformaldehyde, permeabilized with 0.25% Triton, and blocked with 10% goat serum. Primary antibodies used were TUJ1 (1:200, ab78078, Abcam) and GFAP (1:200, 16825-1-AP, Proteintech), followed by secondary antibodies goat anti-mouse 594 fluorescence (A23410, Abbkine) and goat anti-rabbit 488 (A23220, Abbkine). Imaging was conducted using a fluorescence microscope (RVL-100-G, ECHO).

**1.5 Identification of reactive oxygen species (ROS)**

The impact of Mg^2+^ on the viability of cells was assessed using the CCK-8 method and flow cytometry. ENCCs were seeded in 96-well plates at a density of 5000 cells per well and exposed to different doses of MgCl_2_ for 24 hours. Subsequently, 100 μL of 10% CCK-8 solution (Cat. GK10001) was added to each well, followed by incubation for a specified time. Absorbance was measured at 450 nm using a microplate reader. After completing the experimental treatment, cells were collected, washed with pre-cooled PBS, and the cell cycle distribution was assessed. Cells were suspended in PBS at a concentration of 1 × 10^6^ cells/mL. The cell proliferation assay kit (R11056.6) was used for flow cytometry. The cell suspension was centrifuged at 1000 rpm for 5 min. The remaining cells were fixed with 1 mL PBS and 4 mL 95% ethanol at 4°C for 2 h. After centrifugation, the residual cells were exposed to 0.4 mL propidium iodide (PI) reagent at 37 °C in the dark for 30 min. All samples were analyzed using the BD FACS Calibur flow cytometer (BD Biosciences, NJ, USA).

**2.6 NAD(+)/NADH**

To assess NAD(+) and NADH levels and the NAD(+)/NADH ratio, 1 × 10^6^ cells were cultured in neuronal differentiation medium for 7 days. After cell lysis, the supernatant was collected and used for analysis. A NADH standard curve was prepared, and the ethanol dehydrogenase working solution was diluted. Samples were heated at 60 °C for 30 minutes to decompose NAD(+), followed by incubation at 37 °C in the dark to convert NAD(+) to NADH. Absorbance at 450 nm was measured to quantify NAD(+) and NADH levels. Calculations for NAD(+) and NADH levels were performed using specific formulas. This process allowed for the precise quantification of NAD(+) and NADH levels, providing insights into the cellular metabolic state during neuronal differentiation.
